# Seed amplification of released alpha-synuclein strains reveals genotype-specific differences in Parkinson’s disease neurons

**DOI:** 10.3389/fnagi.2026.1917981

**Published:** 2026-09-08

**Authors:** Jessica Chedid, Yuan Tang, Kevin Law, Lachlan Thompson, Yann Gambin, Emma Sierecki, Nicolas Dzamko

**Affiliations:** 1 Macquarie Medical School, Faculty of Health and Human Sciences, Macquarie University, Sydney, NSW, Australia; 2 School of Medical Sciences, Faculty of Medicine and Health, Brain and Mind Centre, University of Sydney, Sydney, NSW, Australia; 3 School of Medical Sciences, Faculty of Medicine and Health, Charles Perkins Centre, University of Sydney, Sydney, NSW, Australia; 4 EMBL Australia Node for Single Molecule Science and School of Biomedical Sciences, Faculty of Medicine, The University of New South Wales, Sydney, NSW, Australia

**Keywords:** alpha-synuclein, IPSC, Parkinson’s disease, seed amplification, strain

## Abstract

Parkinson’s disease is one of a group of diseases collectively termed α-synucleinopathies, all of which are characterized by abnormal accumulation of toxic α-synuclein (α-syn) assemblies which are thought to follow a prion-like propagation model and drive neuronal death. Both intrinsic strain properties and the genetic background of affected individuals are hypothesized to influence α-syn aggregate accumulation, propagation, and extracellular release. As such, the aim of this study is to characterize how different genetic backgrounds modulate α-syn aggregate accumulation and release in response to treatment with different α-syn strains. iPSC-derived neurons from five different groups were employed (healthy non-PD controls (NPC), idiopathic PD (iPD), *GBA1* N370S, *LRRK2* G2019S and *SNCA* A53T) (*n* = 15 cell lines, three lines per group). Individual aggregates in conditioned medium (CM) were measured using a single-particle detection system to quantify released α-syn species and assess strain persistence across genotype groups. Seed amplification assay (SAA) was then applied and linear discriminant analysis (LDA) of single particle properties from CM of α-syn fibril-treated neurons was used to discriminate between groups. α-Syn released into CM showed strain persistence in *LRRK2* G2019S and *GBA1* N370S genotype groups, while *SNCA* A53T neurons exhibited evidence of strain remodeling. Interestingly, amplification-response Δ-feature analysis of single particle properties showed group discrimination beyond either pre- or post- amplification measurements independently, reaching a maximum LOO-CV accuracy of 66.7% (3.3 × chance level) at day 10 post-treatment in CM from fibrils-treated neurons. These findings indicate that genotype-specific cellular responses differentially shape the structural properties of released α-syn in a manner that determines amplification responses, enabling inter-group discrimination at the single-molecule level.

## Introduction

Parkinson’s disease (PD) is the second most common neurodegenerative disorder, affecting approximately 1%–2% of individuals over 65 years of age (de Lau and Breteler, 2006). The disease is characterized clinically by progressive motor symptoms including bradykinesia, resting tremor, rigidity, and postural instability, arising from the selective loss of dopaminergic neurons in the substantia nigra pars compacta (Kalia and Lang, 2015). The pathological hallmark of PD is the presence of Lewy bodies (LBs) and Lewy neurites (LNs), intracellular proteinaceous inclusions found throughout the central and peripheral nervous systems. The major component of these inclusions is aggregated α-synuclein (α-syn), a 140-amino acid presynaptic protein that is abundantly expressed in neurons (Spillantini et al., 1997). While α-syn is normally soluble and involved in synaptic vesicle trafficking and neurotransmitter release (Burré et al., 2010), in PD it misfolds and assembles into insoluble fibrillar aggregates enriched in cross-β-sheet structures (Serpell et al., 2000). The central role of α-syn in PD pathogenesis is further supported by genetic evidence. Multiplications of the SNCA gene (encoding α-syn) cause autosomal dominant PD, with disease severity correlating with gene dosage (Singleton et al., 2003), and point mutations in SNCA, including A53T, A30P, and E46K, lead to early-onset familial forms of the disease (Krüger et al., 1998; Polymeropoulos et al., 1997; Zarranz et al., 2004). Additionally, genome-wide association studies have identified SNCA variants as major risk factors for sporadic PD (Nalls et al., 2014; Simón-Sánchez et al., 2009), establishing α-syn aggregation as a critical pathogenic mechanism across both familial and idiopathic forms of the disease.

Accumulating evidence suggests that α-syn pathology spreads throughout the nervous system via a prion-like mechanism of templated misfolding and cell-to-cell transmission (Brundin et al., 2010; Goedert et al., 2010). Braak et al. (2003) proposed a spatiotemporal staging system for LB pathology in PD, describing a stereotypical caudo-rostral progression from the olfactory bulb and dorsal motor nucleus of the vagus through the brainstem and eventually to cortical regions, correlating with disease progression. This staging pattern implies that α-syn pathology may originate at specific anatomical sites and propagate along neuroanatomically connected pathways. Experimental support for this hypothesis comes from studies demonstrating that intracerebral injection of synthetic α-syn fibrils or brain homogenates from PD patients can induce LB-like pathology, which spreads from the injection site to anatomically connected brain regions over time (Luk et al., 2012; Masuda-Suzukake et al., 2013). Furthermore, post-mortem analysis of PD patients who received fetal mesencephalic transplants revealed LB pathology in grafted neurons years after transplantation, suggesting host-to-graft transmission of pathogenic α-syn (Kordower et al., 2008; Li et al., 2008). At the cellular level, α-syn aggregates can be released from neurons, taken up by neighboring cells, and seed the aggregation of endogenous monomeric α-syn, propagating the pathological cascade (Desplats et al., 2009; Hansen et al., 2011). This templated conversion mechanism, whereby exogenous α-syn fibrils recruit and misfold endogenous α-syn, mirrors the prion paradigm and provides a molecular basis for the observed spreading of pathology in PD.

Similar to prion diseases, where distinct prion strains produce different clinical phenotypes and neuropathological patterns (Collinge and Clarke, 2007), emerging evidence indicates that α-syn can adopt structurally distinct fibrillar conformations, or “strains,” with unique biochemical and pathological properties (Bousset et al., 2013; Peelaerts et al., 2015). Different assembly conditions *in vitro*, including variations in pH, salt concentration, temperature, and the presence of cofactors, can generate α-syn fibrils with distinct morphologies, as revealed by electron and atomic force microscopy (Bousset et al., 2013; Gath et al., 2014). These structural polymorphs or strains exhibit differential stability, seeding efficiency, and cellular toxicity *in vitro*. Cryo-electron microscopy studies have resolved atomic-level structures of α-syn fibrils assembled under different conditions, revealing variations in the arrangement of β-sheet cores (Guerrero-Ferreira et al., 2018; Li et al., 2018). Importantly, distinct α-syn fibril structures have been identified in brain tissue from patients with different synucleinopathies, including PD, dementia with Lewy bodies (DLB), and multiple system atrophy (MSA), suggesting that strain diversity may underlie the phenotypic heterogeneity observed across these diseases (Peng et al., 2018; Schweighauser et al., 2020). The concept of strain fidelity, whereby a particular α-syn conformation is faithfully propagated through templated misfolding, is supported by *in vitro* seeding experiments showing that under certain conditions, fibril polymorphs maintain their structural characteristics through successive rounds of seeded aggregation (Bousset et al., 2013; Guo et al., 2013). However, whether strain fidelity is preserved particularly in neurons harboring disease-associated mutations is less understood. If different genetic backgrounds alter the ability to maintain strain-specific characteristics, this could have important implications for understanding disease heterogeneity and phenotypic variation in PD.

While the effects of individual PD-associated mutations on α-syn aggregation have been extensively studied, how these mutations influence cellular responses to distinct α-syn strains and whether they alter strain fidelity during propagation remain unanswered questions. The *SNCA* A53T mutation, the first identified cause of familial PD, enhances α-syn aggregation propensity and promotes β-sheet formation, leading to accelerated fibril assembly *in vitro* and early disease onset in carriers (Conway et al., 1998; Greenbaum et al., 2005). Mutations in *LRRK2*, most notably G2019S, cause autosomal dominant PD through kinase hyperactivity that disrupts vesicular trafficking, endolysosomal function, and autophagy, potentially affecting the cellular handling of α-syn aggregates (Cookson, 2010; West et al., 2005). Heterozygous mutations in *GBA1*, such as N370S, which encodes the lysosomal enzyme glucocerebrosidase, are the most common genetic risk factors for PD and impair lysosomal degradation pathways critical for α-syn clearance (Mazzulli et al., 2011; Sidransky et al., 2009). Each of these mutations may differentially impact how neurons process, propagate, and potentially modify distinct α-syn strains. Previous work has demonstrated that α-syn fibrils assembled under low-salt conditions “ribbons” versus high-salt conditions “fibrils” exhibit differential seeding capacities and structural properties (Bousset et al., 2013; Gath et al., 2014), making them useful tools for investigating strain-specific responses. However, whether neurons carrying different PD-associated mutations respond differentially to these strains, and critically, whether the genetic background influences the maintenance of strain-specific characteristics during intracellular processing and subsequent release, has not been systematically examined.

Understanding how PD mutations influence strain fidelity is essential for elucidating the mechanisms underlying phenotypic diversity in PD and could inform therapeutic strategies targeting strain-specific pathology. In this study, we generated induced pluripotent stem cell (iPSC)-derived neurons from patients carrying SNCA A53T, LRRK2 G2019S, or GBA1 N370S mutations, as well as from idiopathic PD patients and neurologically healthy controls. We exposed these neurons to structurally distinct α-syn assemblies and assessed the kinetics of intracellular inclusion formation and clearance, the cytotoxicity of these assemblies, the characteristics of released α-syn species, and whether the parental strain signature was maintained or altered during cellular processing. We also used seed amplification of CM to measure induced changes in the single particle fingerprint of released α-syn species as a tool to discriminate between the different experimental groups using individual-molecule data and linear discriminant analysis.

## Materials and methods

### Cell lines

Cell lines used in this study are listed in Table 1. Cell lines with the *LRRK2* G2019S mutation were purchased as fibroblasts from the Coriell biorepository, they have previously been reprogrammed into iPSCs and differentiated to neural stem cells (NSCs) and their differentiation confirmed by immunocytochemistry (Zhao et al., 2020). All other cell lines were obtained as iPSC from the Golub Capital iPSC Parkinson’s Progression Markers Initiative (PPMI) Sub-study^1^, and have been previously described and their differentiation into NSCs and mature neurons characterized (Labrador-Garrido et al., 2023), except for the *SNCA* A53T cells which are characterized as part of this study. The investigators within PPMI contributed to the design and implementation of PPMI and/or provided data and collected samples but did not participate in the analysis or writing of this report. For up-to-date information on the study, visit PPMI-info.org. All work with human iPSC was approved by the University of Sydney human research ethics committee (2017/094) and Macquarie University (#20398).

**TABLE 1:** Cell lines used in this paper were divided into five groups (no PD control, iPD, *LRRK2* G2019S, *GBA1* N370S mutated, and *SNCA* A53T), their identifiers, origins, patient demographics, and mutations are presented in the table.

Group	Cell line	Age	Sex	Mutation	PD diagnosed	Previously characterised (iPSCs, NSCs, neurons)
No PD control (NPC)	PPMI 7428	72	F	–	No	Yes
	PPMI 7521	67	F	–	No	Yes
	PPMI 4035	65	M	–	No	Yes
iPD	PPMI 15733	60	M	–	Yes	Yes
	PPMI 472	59	F	–	Yes	Yes
	PPMI 10106	61	F	–	Yes	Yes
*LRRK2* G2019S	ND 34810	72	M	G2019S	Yes	Yes
	ND 38262	60	M	G2019S	Yes	Yes
	ND 30244	73	M	G2019S	Yes	Yes
*GBA1* N370S	PPMI 17880	62	M	N370S	Yes	Yes
	PPMI 5484	59	F	N370S	Yes	Yes
	PPMI 6745	62	M	N370S	Yes	Yes
*SNCA* A53T	PPMI 11555	36	M	A53T	Yes	No
	PPMI 11556	54	F	A53T	Yes	No
	PPMI 11557	53	M	A53T	Yes	No

### Generation of human neural stem cells (NSCs) from iPSCs carrying SNCA A53T mutation

*SNCA* A53T iPSC were cultured in 6-well plates at 37 °C in a humidified incubator with 95% air and 5% CO_2_. For neural induction, iPSCs were cultured in Neurobasal medium (Gibco, 21103049) supplemented with 1/50 neural induction supplement (Gibco, A1647701) and 1× penicillin-streptomycin (Gibco, 15140122), with medium replaced every 2 days. On day 7, passage 0 (P0) NSCs were washed with 2 mL Ca^2+^/Mg^2+^-free DPBS (Gibco, 14190144) and detached using 1 mL pre-warmed StemPro Accutase Cell Dissociation Reagent (Gibco, A1110501) at 37 °C for 5–8 min. Detached cells were harvested with a cell scraper (Thermo Fisher Scientific, 08-771-1A), transferred to a 15 mL conical tube, and residual cells were collected by rinsing each well with 1 mL DPBS. The cell suspension was filtered through a 100 μm strainer (Thermo Fisher Scientific, 08-771-19), centrifuged at 300 × *g* for 4 min, and the supernatant was discarded. Cells were resuspended in 2 mL DPBS, centrifuged again at 300 × *g* for 4 min, and resuspended in Neural Expansion Medium (NEM) composed of 50% Advanced DMEM (Gibco, 12491015), 50% Neurobasal medium, supplemented with 1/50 neural induction supplement and 1 × penicillin-streptomycin, for subsequent culture.

### Pre-coating of 96-well plates for neuronal differentiation

For neuronal differentiation, 96-well plates (PerkinElmer, #6055300) were double-coated with either 20 μg/mL poly-L-ornithine (Thermo Fisher Scientific, #P4957) and 10 μg/mL laminin (Thermo Fisher Scientific, #23017015) or 100 μg/mL poly-D-lysine (Thermo Fisher Scientific, #A3890401) and 15 μg/mL laminin (Thermo Fisher Scientific, #23017015). The plates were coated with Poly-L-ornithine or poly-D-lysine at 37 °C for at least 1 h followed by washes and Laminin coating at 37°C for at least 1 h. Coated plates were sealed with parafilm and stored at 4 °C for up to 1 week. The laminin solution was removed immediately before adding cells to prevent the wells from drying.

### NSC culture and neuronal differentiation

Human NSCs from all groups were cultured in NEM (at 37 °C in a humidified 95% air, 5% CO_2_ incubator), with medium replaced every 2 days. For propagation, NSCs were maintained in 6-well plates, detached using 1 mL StemPro Accutase Cell Dissociation at 37 °C for 3–5 min and re-plated in NSC culture medium with 10 μL/mL RevitaCell supplement (Gibco, A2644501). For differentiation and experiments, NSCs were seeded in 96-well plates at 1.5 × 10^4^ cells/well in poly-L-ornithine- and laminin-coated 96-well plates and incubated overnight in culture medium. Differentiation into neurons was induced using neural differentiation medium (NDM), which consisted of Neurobasal medium supplemented with 2% B-27 serum-free supplement (Gibco, 17504044), 1% GlutaMAX (Gibco, 35050061), and 1% penicillin-streptomycin. The medium was replaced every 2 days, with half of the volume replaced each time. After 5 days, the medium was switched to neural maturation medium (NMM), which was composed of Neurobasal Plus medium (Gibco, A3582901) supplemented with 2% B-27 Plus supplement, 1% GlutaMAX, and 1% penicillin-streptomycin, with half the medium replaced every 2 days for 15 days in total.

### Characterisation of SNCA A53T NSCs and neurons

To compare the differences in marker expression in *SNCA* A53T cells before and after neural induction, one batch of iPSCs was fixed before undergoing neural induction. The other batch of iPSCs was incubated with neural induction medium for 7 days to generate NSCs. Based on previous findings, OCT-4A, a transcription factor expressed in the nucleus, is restricted to pluripotent and germ line cells (Schöler et al., 1989; Schoorlemmer and Kruijer, 1991). Meanwhile, the cytoplasmic protein Nestin is expressed primarily in neural stem cells and progenitor cells (Krupkova et al., 2011). Therefore, iPSCs before and after neural induction were stained for OCT-4A (Cell Signalling, 2840, 1:500) and Nestin (Abcam, ab22035, 1:500) to determine whether NSCs had been successfully generated. To confirm differentiation of NSCs into mature neurons, a batch NSC were seeded into 96-well plates and incubated for 2 days before being stained for Nestin, while a second batch was treated with differentiation medium for 14 days for neuronal differentiation before being stained for Microtubule-Associated Protein 2 (MAP2) (ThermoFisher, PA110005, 1:500), a marker widely used to identify neurons (DeGiosio et al., 2022). All three cell lines were successfully differentiated into NSCs (Supplementary Figure 1) and then into mature neurons (Supplementary Figures 2A–C) that expressed MAP2 in at least 80% of cells (Supplementary Figures 2D, E) consistent with the percentage of MAP2 expressing cells in all other experimental lines/groups.

### Generation and seeding of ribbons and fibrils α-syn assemblies

**FIGURE 1:**
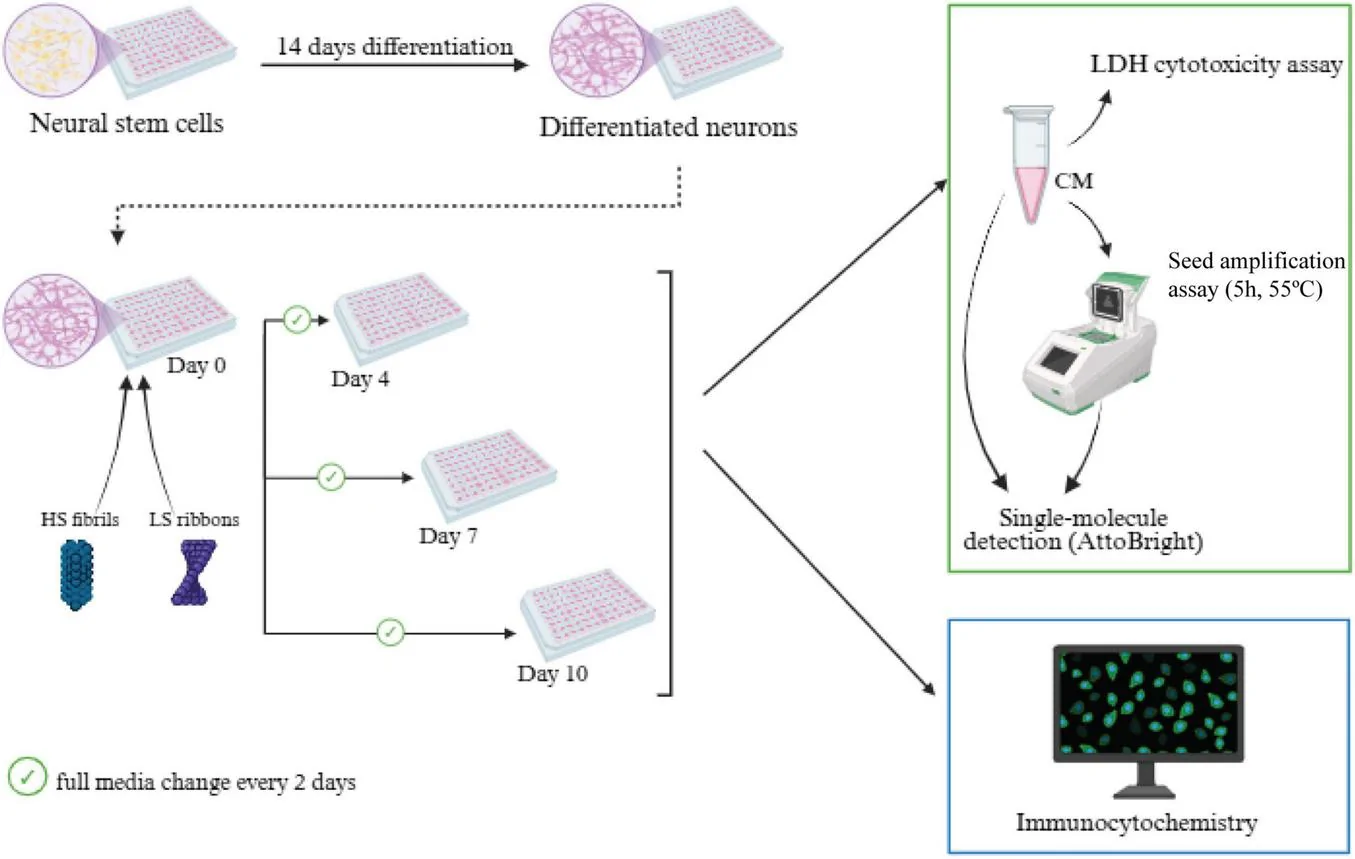
Experimental workflow of the study. iPSC-derived neural stem cells were differentiated for 14 days into mature neurons. Differentiated neurons were treated with either α-syn HS “fibrils” or α-syn LS “ribbons” at 5 μg/ml. The neurons were maintained in culture for 4, 7, or 10 days with full media change performed at day 2 to remove exogenous fibrils, followed by half-media changes every 2 days starting day 4 for the longer timepoints. At each of the experimental timepoints, the cells were fixed for immunocytochemistry, and the CM was collected and stored for LDH cytotoxicity assay and single-molecule detection using the Attobright system. Single molecule detection was performed on the CM directly or after amplification for 5 h at 55 °C to assess the seeds amplification capabilities.

The generation of ribbon and fibril assemblies using full length human α-syn prepared in low- and high-salt buffers has been previously published (Lau et al., 2024). Briefly, purified recombinant human wild-type α-syn was buffer-exchanged into low-salt buffer (LSB 5 mM Tris, pH 7.5, 0.02% w/v NaN_3_) using Zeba spin columns (ThermoFisher, #89890). Low-salt “ribbons” and high-salt “fibrils” were prepared in screw-top microtubes (SSIbio, 2340-00), secured with caps (SSIBio, 2001-00) with a final volume of 600 μL. Low-salt ribbons were generated by diluting α-syn to 250 μM in LSB. High-salt fibrils were generated by diluting α-syn to 250 μM in high-salt buffer (HSB; 50 mM Tris, pH 7.5, 150 mM KCl, 0.02% w/v NaN_3_). Tubes were agitated horizontally at 600 rpm and 37 °C in a Falcon tube secured to a vortexer. Ribbons and fibril assemblies were collected after 7 and 2 days, respectively, and sonicated using an M220 focused-ultrasonicator (Covaris, 500295) at 12 °C with 75 W peak power, 25% duty factor, 200 cycles/burst, 5 s delay, and 60 cycles, then flash-frozen in liquid nitrogen and stored at −80 °C as single-use aliquots. For subsequent use, samples were thawed and maintained at room temperature to prevent disassembly. Before cell treatment, assemblies were diluted to 0.1 mg/mL in sterile DPBS, sonicated (120 s, 40% amplitude, 1 s on/off pulses; Q125, QSonica) to generate PFFs and further diluted to working concentrations in pre-warmed culture medium. Assemblies were prepared and applied to cells within 15 min of thawing. For seeding experiments, differentiated neurons were treated with 5 μg/mL of ribbons or fibrils on Day 0. Cells were washed with PBS for three times, followed by the addition of fresh medium to remove residual exogenous α-syn assemblies on Day 2. On Days 4, 7, and 10 post-seeding, conditioned medium (CM) was collected, centrifuged at 300 × *g* for 5 min, then snap-frozen and stored at −80 °C. Cells were then fixed using 4% paraformaldehyde for immunocytochemistry and imaging. A half medium change was performed at days 4 and 6 for the 7 days timepoint, and at days 4, 6, and 8 for the 10 days timepoint. An overview of the experimental design is shown in Figure 1. The ribbons and fibrils materials used in this study are the same preparations used in the previously published manuscript by Lau et al. (2024) and have been thoroughly characterized. Transmission electron microscopy (TEM) images of both ribbons and fibrils species generated are included in Supplementary Figure 3 highlighting structural differences between the two types of seeds.

### Cellular toxicity

Lactate dehydrogenase (LDH) was measured in culture medium using the CytoTox96 Non-Radioactive Cytotoxicity Assay (Promega, G1780) according to the manufacturers’ instructions. Medium was collected from cells during medium changes at specified timepoints and stored at −80 °C until analysis. To determine maximum LDH activity, control wells containing cells alone were lysed using the provided lysis solution. CytoTox96 Reagent was prepared by combining 12 mL assay buffer with substrate mix immediately before use. In triplicate, 50 μL of culture medium was added to a 96-well transparent plate (Greiner Bio-One, 65101) and mixed with 50 μL CytoTox96 Reagent. Samples were incubated at room temperature for 30 min, protected from light. After incubation, 50 μL Stop Solution was added to each well, and absorbance was measured at 492 nm within 1 h using a Tecan Infinite M1000 plate reader (Tecan, 30034301). Background absorbance was determined from three wells containing a medium blank (cell-free medium) per plate, and their mean value was subtracted from sample readings. Percent cytotoxicity was calculated as the ratio of sample LDH release to maximum LDH release from lysed cells.

### Confocal microscopy

Neurons differentiated in 96-well plates were washed with PBS (ThermoFisher, 18912014) and fixed with 4% w/v paraformaldehyde (Sigma Alrich, 158127-3KG) for 20 min. After washing again with PBS, cells were permeabilized with 0.3% v/v triton (Sigma Alrich, X100-500ML) for 15 min. Blocking was performed with 3% w/v BSA (Sigma Alrich, A9647-1KG) and 0.1% v/v Triton for 1 h. The primary antibodies used are mouse anti-total α-syn (BD Biosciences, 610787) diluted to 1:300 and chicken anti-MAP2 (ThermoFisher, PA110005) diluted to 1:500, both diluted in 3% w/v BSA and 0.1% v/v triton (blocking buffer) and incubated overnight at 4 °C. After washing with PBS, donkey anti-mouse Alexa Fluor 488 (Invitrogen, A21202) and donkey anti-chicken Alexa Fluor 647 (Jackson ImmunoResearch Laboratories, 703-605-155) were added for 1 h in the dark at 1:500 dilution in blocking buffer. Following another wash with PBS, DAPI (Sigma Alrich, D9542-5MG) was added to the last wash at a 1:10,000 dilution and incubated for 15 min. Z-stacks were taken using a Nikon C2 confocal microscope at 40 × magnification.

### Image analysis

Images were analyzed using ImageJ. Maximum intensity projection of z-stacks was performed for each image, and the threshold tool was used to highlight stained areas and measure integrated density and area. Cell number was counted manually based on DAPI staining. Cells with nuclei touching to the field of view (FOV) edges were excluded from analysis. Three images (80–280 cells per image) were analyzed per cell line/timepoint/treatment condition. α-syn inclusions area was normalized to DAPI count.

### Seed amplification assay using conditioned media

Conditioned medium collected at different timepoints was diluted as follows: 8 μL of the CM was added to 32 μL of the substrate mix, resulting in a 40-μL reaction mix (1x PBS, 10 μM Thioflavin T (Sigma Aldrich, T3516), 20 μM α-syn K23Q monomers) for each sample. One half of the reaction mix was directly analyzed by AttoBright single molecule spectroscopy, and the other was amplified in a PCR machine before AttoBright analysis resulting in a “direct” and “amplified” reading for each sample. The amplified portion was incubated at 55 °C for 5 h in a Mastercycler X50s PCR machine (Eppendorf, 6306000061). α-syn K23Q monomers were used as the monomer template to reduce non-specific signal from self-aggregation and prepared as previously described (Groveman et al., 2018).

### Single particle spectroscopy analysis

**FIGURE 2:**
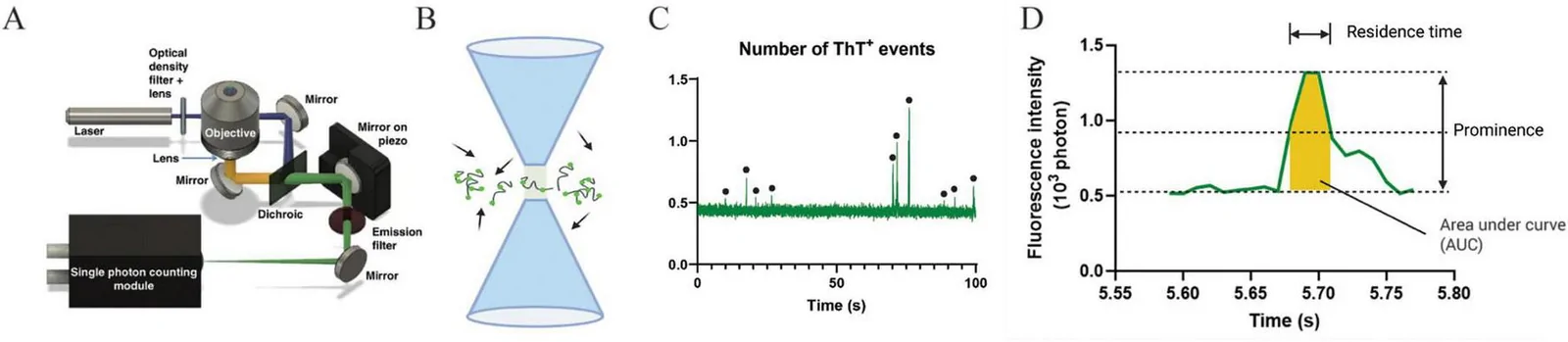
Detection of individual ThT-positive aggregates using single molecule counting. **(A)** Overview of optical path. The design utilizes a single excitation laser, which is focused to a very small (femtolitre) observation volume using a water-immersion objective (Zeiss × 40 or × 63, 1.2 NA). The emitted fluorescence is separated from the excitation fluorescence using a dichroic mirror and further filtered before detection by a single photon avalanche photodiode (SPAD, Micro Photon Devices). **(B)** Principle of single molecule detection: a very small (femtolitre) detection volume coupled to single photon detectors can measure freely diffusing single particles with high sensitivity as they diffuse in and out of the confocal volume. **(C)** Single molecule reading of fluorescence intensity over time shows a constant ThT background with single peaks corresponding to single molecules diffusing through the detection volume. **(D)** Schematic of quantifiable properties of peaks: prominence, residence time, and area under the curve (AUC).

For single molecule particle post the seed amplification, CM samples prepared as described in the previous paragraph were transferred from PCR tubes to a customized polydimethylsiloxane (PDMS) plate attached to a glass coverslip (ProSciTech, G425-4860) and imaged using an inverted 3D-printed AttoBright confocal microscope as previously described (Brown et al., 2019). Traces were recorded for up to 5 min at room temperature (∼450 μW laser power), with 100-s traces binned at 10-ms intervals, generating up to three fluorescence spectroscopy traces per sample. Analysis of fluorescence traces was performed using a Python script, evaluating each trace for the total number of ThT+ events per measurement, as well as the prominence, residence time, and area under the curve of individual peaks (Figure 2). The Python script used for analysis is included as a Supplementary file.

### Data analysis

Single-molecule fluorescence measurements of CM yielded three peak morphology features per detected aggregate: peak height (ThT fluorescence intensity, proportional to amyloid density), peak width (transit time through the detection volume, proportional to hydrodynamic radius), and peak area (integrated ThT signal, mix of number of ThT and quantum efficiency for each ThT). Multivariate outliers were identified and removed within each analysis subgroup (defined by genotype group and timepoint) using the Mahalanobis distance criterion. A peak was excluded if its Mahalanobis distance in log-transformed feature space exceeded the threshold *d* = √χ^2^0.975(df = 3) = 3.06, corresponding to the 97.5th percentile of a chi-squared distribution with three degrees of freedom. This threshold was applied independently to each group-by-timepoint subgroup to account for subgroup-specific covariance structure. Principal component analysis (PCA) was applied to the standardized log-transformed feature matrix to characterize the primary axes of variation in single-molecule peak morphology and to visualize relationships among aggregate populations. PCA was performed separately for the unamplified and amplified datasets. PCA biplots display loading vectors alongside individual peak scores colored by genotype group, with 95% confidence ellipses per group computed from the bivariate normal distribution of PC1 and PC2 scores. Linear discriminant analysis (LDA) was used to identify the linear combination of peak morphology features that maximally discriminated among the five genotype groups. LDA was applied separately to each timepoint and to the pooled dataset, using the three log-transformed, standardized features (height, width, area) as inputs. Equal prior probabilities were assumed across groups, consistent with the balanced design (*n* = 3 biological replicates per group). The solver used singular value decomposition without regularization. Up to four discriminant functions were extracted (*n* classes−1); the first two discriminant axes (LD1, LD2) are displayed in score plots with group-level confidence ellipses. Classification performance was quantified using leave-one-out cross-validation (LOO-CV) at the individual peak level, where each peak was classified using an LDA model trained on all remaining peaks. LOO-CV accuracy is reported as the proportion of peaks correctly assigned to their true genotype group (chance level: 20% for five equally sized groups). To assess the statistical significance of the observed accuracy, a permutation test was performed: group labels were randomly shuffled 1,000 times, LDA was re-run on each permuted dataset using the same LOO-CV procedure, and the permutation *p*-value was calculated as the proportion of null accuracies meeting or exceeding the observed value. Because individual peaks within a sample are not statistically independent, a complementary sample-level analysis was performed in which peak features were averaged within each biological replicate before LDA, yielding *n* = 15 data points (5 groups × 3 replicates). LOO-CV on these sample-level summaries provides a conservative estimate of group discriminability that is not inflated by within-sample peak correlations. To test whether the multivariate centroid of peak morphology features differed significantly among genotype groups, a one-way multivariate analysis of variance (MANOVA) was performed on log-transformed, standardized features. Pillai’s trace was used as the test statistic, chosen for its robustness to violations of multivariate normality and unequal group sizes relative to Wilks’ lambda. Analyses were performed separately for each timepoint and for the pooled dataset. To complement MANOVA with a permutation-based test that makes no distributional assumptions, a permutational multivariate analysis of variance (PERMANOVA) was applied to the standardized log-transformed feature matrix. Pairwise squared Euclidean distances were computed among all peaks within each analysis subset, and a pseudo-F statistic was calculated as the ratio of between-group to within-group sums of squared distances, weighted by group size. Statistical significance was assessed by comparing the observed pseudo-F to a null distribution generated by 999 random permutations of group labels. PCA and LDA scatter plots were generated in Python (version 3.14) using the scikit-learn library. Raw peak morphology features (height, width, and area) were log-transformed, and z-score standardized prior to analysis figures were rendered using Matplotlib. All other data were analyzed using Two-way ANOVA (CM peaks counts and asyn area per cell) with Dunnett’s *post-hoc* multiple comparisons test. All remaining figures were generated using GraphPad Prism (version 10, GraphPad Software, Boston, MA, USA).

## Results

### Significant differences in the number of ThT-positive events in CM captured by single-particle detection of fibril and ribbon-treated neurons across genotypes and time

**FIGURE 3:**
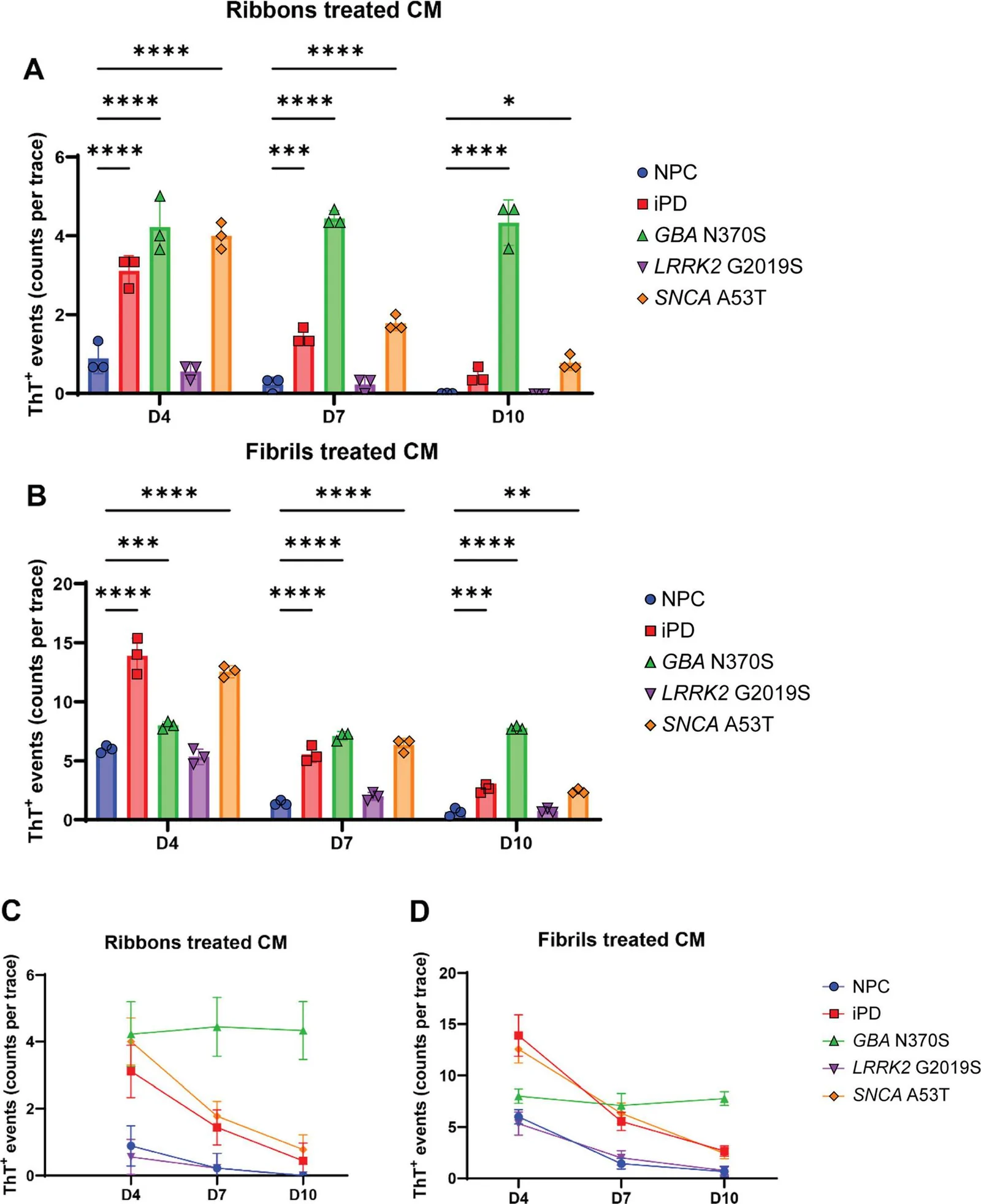
Direct reading of CM collected in seeding experiments. Differentiated iPSC-derived neurons were treated with 5 μg/mL of “ribbons” and “fibrils.” The CM was collected on Day 4, 7, and 10 after the treatment. The CM was added in a PBS solution containing 10 μM ThT and 20 μM K23Q α-syn monomers. The solution was directly read on Attobright. Bar graphs show ThT-positive events detected per trace in CM treated with “ribbons” **(A)** and “fibrils” **(B)**. Line charts show the decline of ThT-positive events in CM treated with “ribbons” **(C)** and “fibrils” **(D)**. NPC is in blue; iPD is in red, N370S is in green, G2019S is in purple, A53T is in orange. Data are presented as mean ± SD in the graph (*n* = 3 biological replicates, each representing the average of three technical replicates). Statistics used ordinary two-way ANOVA. For all graphs, *P < 0.05, **P < 0.01, ***P < 0.001, ****P < 0.0001.

Differentiated iPSC-derived neurons were treated with either α-syn “fibrils” or “ribbons” at 5 μg/ml, maintained in culture for 4, 7, or 10 days. CM from seeding experiments was collected at each of these timepoints and analyzed for ThT-positive detection using Attobright. Direct reading (without amplification) of CM from either “ribbons” (Figure 3A) or “fibrils” (Figure 3B) treated neurons showed that the number of ThT-positive events from iPD, *GBA1* N370S, and *SNCA* A53T neurons were significantly higher than that of NPC neurons on Day 4 and 7. At day 10, CM of “ribbons” treated neurons maintained a higher number of ThT-positive events for *GBA1* N370S and *SNCA* A53T neurons only (Figure 3A). In the CM from “fibrils” treated neurons at day 10, the number of ThT-positive events remained higher than the control for iPD, *GBA1* N370S and *SNCA* A53T (Figure 3B). In contrast to the other disease mutation groups, the number of ThT-positive events in *LRRK2* G2019S CM was not significantly different from control at any timepoint for either “ribbons” (Figure 3A) or “fibrils” (Figure 3B) treated neurons. Time course analysis showed that ThT-positive events gradually decreased over time in CM for all lines except *GBA1* N370S neurons, which maintained a stable number of ThT-positive events at all the examined timepoints regardless of being treated with “ribbons” (Figure 3C) or “fibrils” (Figure 3D) as was confirmed by 2-way ANOVA with Dunnett’s *post-hoc* multiple comparisons test showing a significant decrease in the number of ThT events in all groups except *GBA* N370S between day 4 and day 7 on one hand, and day 4 and day 10 on the other hand for fibrils and ribbons treated cells (Dunnett’s *P*-values: fibrils-treated: NPC: d4–d7: *P* < 0.0001, d4–d10: *P* < 0.0001, iPD: d4–d7: *P* < 0.0001, d4–d10: *P* < 0.0001, *GBA* N370S*:* d4–d7 *P* = 0.147, d4–d10: *P* = 0.726, *LRRK2* G2019S: d4–d7: *P* < 0.0001, d4–d10: *P* < 0.0001, *SNCA* A53T: d4–d7: *P* < 0.0001, d4–d10: 0.0001. Ribbons-treated: NPC: d4–d7: *P* = 0.0395, d4–d10: *P* = 0.004, iPD: d4–d7: *P* = 0.0019, d4–d10: *P* = 0.0002, *GBA* N370S*:* d4–d7 *P* = 0.878, d4–d10: 0.912, *LRRK2* G2019S: d4–d7: *P* = 0.317, d4–d10: *P* = 0.024, *SNCA* A53T: d4–d7: *P* < 0.0001, d4–d10: 0.0001). We note that there was one other non-significant change in the *LRRK2* G2019S ThT events in ribbon treated cells between days 4 and 7 (*P* = 0,317), however, the decrease was significant between days 4 and 10 (*P* = 0.024) unlike *GBA* N370S where there was no decrease across all three timepoints.

### α-syn species released from SNCA A53T neurons into CM show distinctive amplification responses compared to all other groups

**FIGURE 4:**
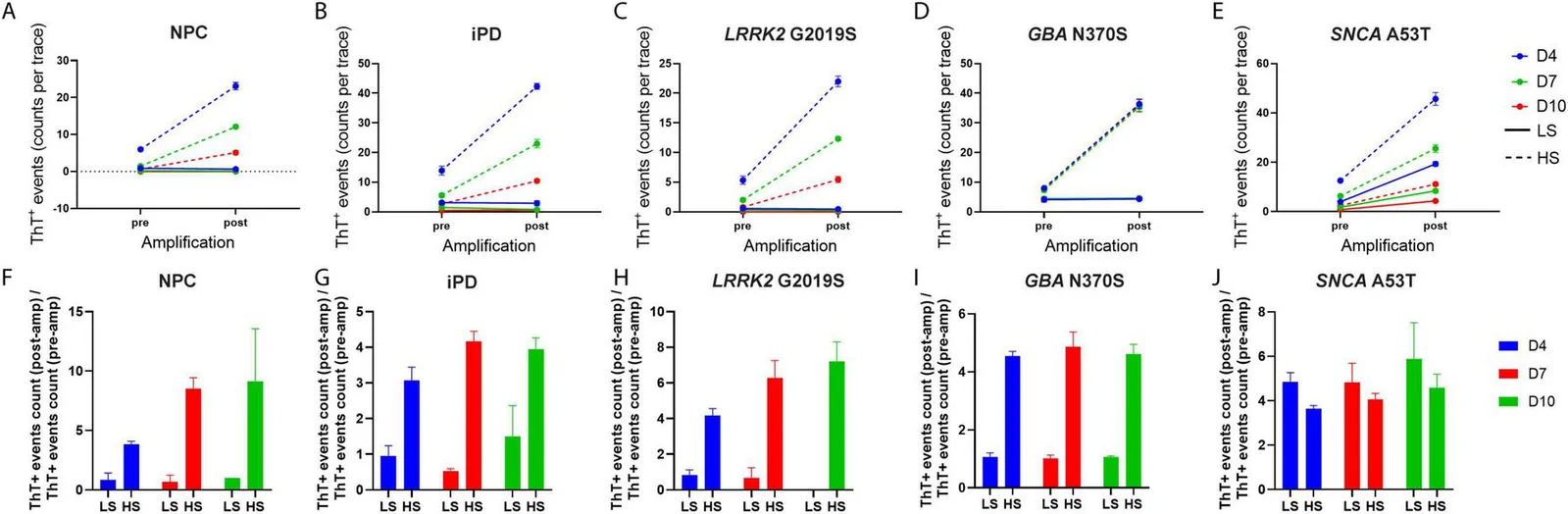
Direct and amplified readings of CM collected from seeding experiments. CM collected at days 4, 7, and 10 was added to solution of PBS containing 10 μM ThT and 20 μM K23Q α-syn monomers. The solution was read directly on Attobright or amplified at 55 °C for 5 h then read on the Attobright. Line charts show the number of ThT-positive events detected per trace before and after amplification from CM collected at different timepoints (4 days in blue, 7 days in green, and 10 days in red) from neurons treated with “fibril” or “ribbons” (“ribbons” in dotted lines and “fibrils” solid lines) **(A–E)**. Bar graphs show the data transformed as a ratio of ThT-positive events post- amplification/ThT-positive events pre-amplification for each treatment condition (“fibrils” or “ribbons”) at each timepoint (4, 7, or 10 days) **(F-J)**. Data are represented as mean ± SD in the graph (*n* = 3 biological replicates, each representing the average of three technical replicates).

To investigate the amplification potential of α-syn species released from neurons of different groups treated with either “ribbons” or “fibrils,” we subjected the collected CM to seed amplification in the presence of α-syn monomer at 55 °C for 5 h. Single-particle detection was then used to quantify the number of ThT-positive events. The data showed that NPC (Figure 4A), iPD (Figure 4B), *LRRK2* G2019S (Figure 4C), and *GBA1* N370S (Figure 4D) “ribbons” treated neurons released α-syn species that do not readily amplify under these conditions, as shown by the absence of increase in the number of ThT-positive events in the CM samples post-amplification. This was not surprising since the amplification conditions used preferentially induce amplification of fibril-like species rather than ribbons-like species (Lau et al., 2024). Indeed, CM from NPC (Figure 4A), iPD (Figure 4B), *LRRK2* G2019S (Figure 4C), *GBA1* N370s (Figure 4D), and *SNCA* A53T (Figure 4E) treated with “fibrils” showed high amplification potency, revealed by an increase in the number of ThT-positive events. Unexpectedly, the CM of “ribbons” treated *SNCA* A53T neurons exhibited similar amplification characteristics to CM from “fibrils” treated neurons (Figure 4E). The ratio of the number of ThT-positive events post-amplification/pre-amplification was calculated for each group × timepoint to control for the variability in the initial number of events pre-amplification in the comparison. The same *SNCA* A53T specific result was observed in the ratio data, where “ribbons” treated neurons had a similar ratio to the “fibrils” treated neurons in the *SNCA* A53T group only (Figure 4J) unlike NPC (Figure 4F), iPD (Figure 4G), *LRRK2* G2019S (Figure 4H), and *GBA1 N370S* (Figure 4I). This demonstrates a potential exception to the “strain fidelity” observed in the α-syn species secreted from other mutation groups, where “ribbons” treated neurons consistently released α-syn species with low amplification potential in the CM, as opposed to the highly amplifiable species released by HS “fibrils” treated neurons.

### Linear discriminant analysis of peak features in unamplified and amplified CM allows some distinction between groups

Next, we aimed to characterize and identify discriminatory single-particle features of the ThT-positive events in the CM. This was done only in the original and amplified CM of neurons treated with “fibrils” because, as described above, “ribbons” treated neuron CM was not consistently amplified in all groups, resulting in a very small sample size. As such, we examined the data from “fibrils” treated neurons on an individual peak level (*n* = 739 unamplified peaks, *n* = 3108 amplified peaks). Following natural log-transformation and Mahalanobis-distance outlier removal, in the unamplified dataset, 31 of 739 peaks were excluded (4.2%); in the amplified dataset, 115 of 3,108 peaks were excluded (3.7%). Following outlier removal, features within each dataset were standardized to zero mean and unit variance (z-score normalization) using parameters estimated from the full dataset. Prior to analysis, raw peak features showed strong positive skewness (skewness 3.7–6.2); log-transformation reduced skewness to 0.4–1.0, assessed by Shapiro-Wilk normality testing within each group-by-timepoint subgroup.

**FIGURE 5:**
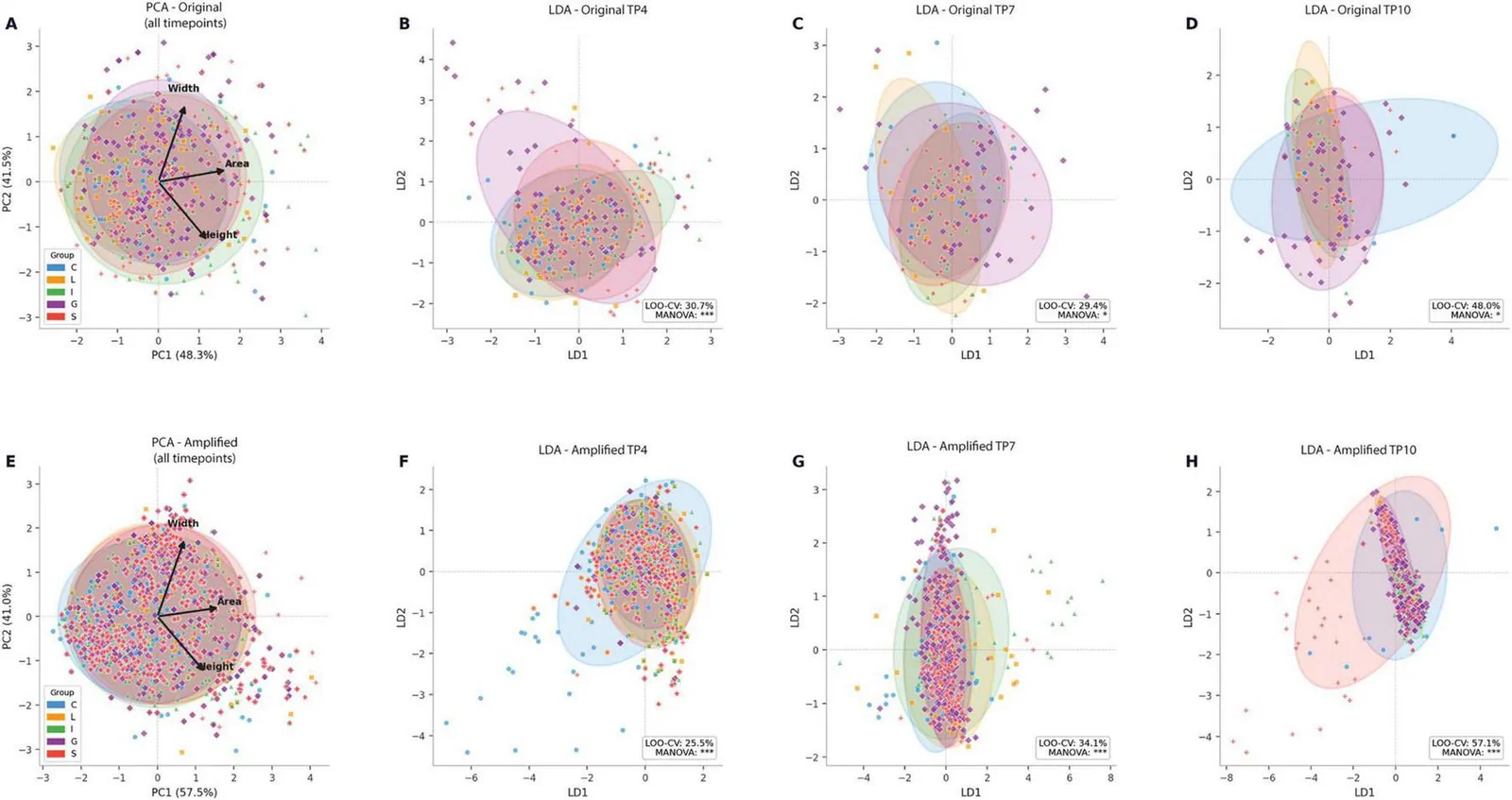
Principal component analysis and linear discriminant analysis of single-molecule peak morphology features in unamplified and amplified CM. Single-molecule ThT fluorescence measurements were performed on conditioned medium (CM) collected from iPSC-derived neurons carrying five genotypic backgrounds — control (C), *GBA1* N370S (G), iPD (I), *LRRK2* G2019S (L), and *SNCA* A53T (S) — at days 4, 7, and 10 post-fibril treatment (*n* = 3 biological replicates per group). Peak morphology features (height, width, and area) were log-transformed, Mahalanobis-distance outlier-removed (χ^2^0.975, df = 3), and z-score standardized prior to analysis. **(A)** PCA score plot of unamplified CM pooled across all timepoints (*n* = 708 peaks after outlier removal). PC1 explained 48.3% of total variance (dominated by peak height and area loadings, reflecting amyloid content) and PC2 explained 41.5% (dominated by peak width versus height contrast, reflecting aggregate compactness). Shaded ellipses represent 95% confidence regions per group. **(B–D)** LDA score plots of unamplified CM at days 4 **(B)**, 7 **(C)**, and 10 **(D)**. LDA was performed on log-transformed, standardized features with group labels as the supervised criterion. Leave-one-out cross-validated (LOO-CV) peak-level classification accuracy and MANOVA significance (Pillai’s trace) are annotated per panel. Shaded ellipses represent 95% confidence regions per group. Replicate identifiers are shown for each peak cluster. **(E)** PCA score plot of amplified CM pooled across all timepoints (*n* = 2,993 peaks after outlier removal). PC1 explained 57.5% of variance and PC2 41.0%, with qualitatively unchanged loading structure relative to the unamplified dataset. **(F–H)** LDA score plots of amplified CM at days 4 **(F)**, 7 **(G)**, and 10 **(H)**. SAA amplification was performed for 5 h prior to single-molecule detection. LOO-CV accuracy and MANOVA significance are annotated per panel. MANOVA significance: *p < 0.05, ***p < 0.001. PCA, principal component analysis; LDA, linear discriminant analysis; LOO-CV, leave-one-out cross-validation; MANOVA, multivariate analysis of variance.

Firstly, principal component analysis was performed on the transformed data. In the unamplified dataset, the first two principal components explained 48.3% and 41.5% of total variance (Figure 5A). PC1 was dominated by height and area loadings (0.557 and 0.772, respectively), reflecting overall amyloid content per aggregate, while PC2 contrasted width (loading: 0.790) against height (loading: −0.601), representing a compactness axis along which wider, lower-density aggregates are distinguished from narrower, denser ones. A consistent feature structure was observed in the amplified dataset (PC1: 57.5%, PC2: 41.0), indicating that SAA amplification expands the dynamic range of the features while preserving their structural relationships (Figure 5E).

Next, group separability was quantified by performing linear discriminant analysis (LDA). LDA was applied to the unamplified CM dataset to determine whether unamplified peak morphology alone could discriminate among the five genotype groups. Examining individual timepoints revealed a temporal pattern in discrimination. At Day 4, separation was strongest (LOO-CV: 30.7%; MANOVA *p* < 0.001; 6/10 pairs significant), reflecting the initial heterogeneity in how genotype groups respond to exogenous fibril seeding (Figure 5B). Discrimination weakened substantially at Day 7 (LOO-CV: 29.4%; MANOVA *p* = 0.026; 1/10 pairs significant), suggesting convergence of aggregate populations secreted during the intermediate medium collection window (Figure 5C). At Day 10, LOO-CV accuracy rose to 48.0%, but MANOVA significance was only marginal (*p* = 0.010), and pairwise testing identified only one significant pair (iPD vs *SNCA* A53T) (Figure 5D). The discrepancy between LOO-CV accuracy and MANOVA significance at Day 10 likely reflects the small peak count at this timepoint (*n* = 123) and high within-group variance, rather than strong multivariate centroid separation. Pairwise Mann-Whitney U tests on LDA scores (BH-corrected) identified six significant group pairs across the pooled dataset, with the most consistent separation between *LRRK2* G2019S and iPD (*p* < 0.01) and between control and iPD (*p* < 0.01) (Supplementary Figure 4).

Next, the amplified peaks dataset was examined and revealed enhanced and temporally evolving group separation: At Day 4 (*n* = 1,473 amplified peaks), discrimination was modest (LOO-CV: 25.5%; 5/10 pairs significant (Figure 5F). At Day 7 (*n* = 926), discrimination was modestly increased (LOO-CV: 34.1%; 2/10 pairs) (Figure 5G). The most striking discrimination emerged at Day 10 (*n* = 594 amplified peaks). LOO-CV accuracy reached 57.1%, 2.9 times the chance level, and MANOVA Pillai’s trace increased substantially to 0.307 (*p* < 0.001) (Figure 5H). Pairwise testing at this timepoint identified 7 of 10 group pairs as significantly separated (Supplementary Figure 4). iPD was the most distinctly positioned genotype, separating significantly from all four other groups: control (*p* < 0.01), *LRRK2* G2019S (*p* < 0.001), *GBA1* N370S (*p* < 0.001), and *SNCA* A53T (*p* < 0.001). *GBA1* N370S and *SNCA* A53T were also mutually separable (*p* < 0.001), and *SNCA* A53T differed from control (*p* < 0.05). Notably, the *LRRK2* G2019S group did not separate significantly from control or *GBA1* N370S at Day 10. Peak-level LOO-CV accuracy figures should, however, be interpreted with the caveat that individual peaks within the same biological replicate are not independent observations, sharing the same cell population and handling conditions. Peak-level accuracy is therefore likely inflated relative to true between-sample discriminability, which is addressed by the sample-level analysis (*n* = 15) reported below.

### Amplification-response change in single particle features significantly improves the group discrimination at sample level using LDA

To test whether the structural response to SAA amplification, rather than either pre- or post-amplification peak morphology observed separately, carries genotype-specific information, we computed, for each biological replicate at each timepoint, the amplification delta (Δ) for each feature: the difference between mean log-transformed amplified and unamplified peak values [Δ = log(amplified)−log(unamplified); see section “Materials and methods”]. This metric isolates the fold-change in structural properties induced by the amplification step, providing a measure of aggregate seeding potency that is normalized to each sample’s own baseline.

**FIGURE 6:**
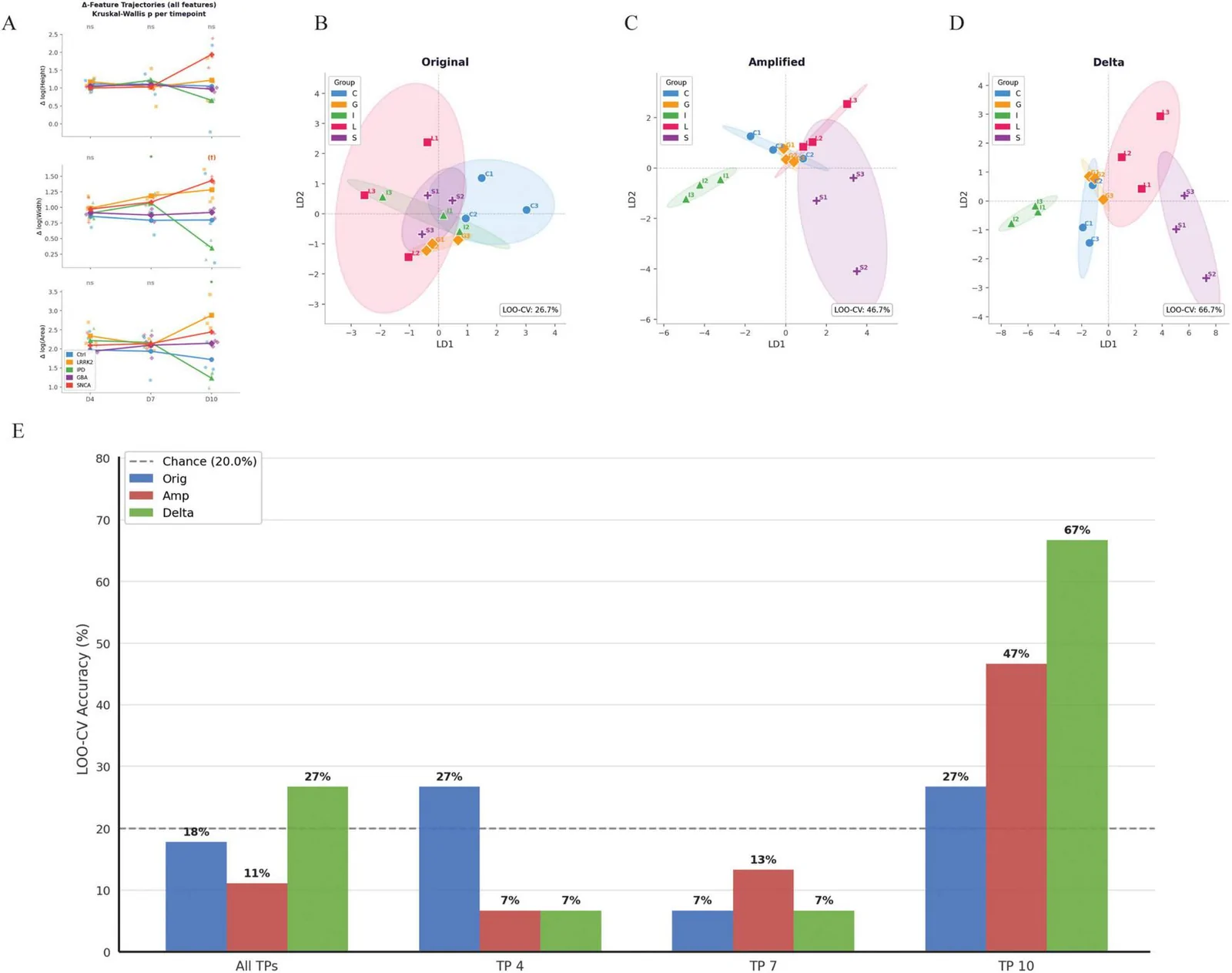
Amplification-induced change in peak morphology features. Δ-features were computed per biological replicate as the difference between mean log-transformed amplified and unamplified peak values [Δ = log(amplified)–log(unamplified)]. **(A)** Trajectory plots of Δ-feature values for all three peak morphology features (Δ log(Height), Δ log(Width), Δ log(Area), top to bottom) across days 4, 7, and 10, shown per genotype group (*n* = 3 replicates per group per timepoint). Lines connect group means; individual replicate values are overlaid as points. Kruskal-Wallis H-test significance for between-group differences in Δ-feature magnitude is annotated above each timepoint. **(B–D)** Sample-level LDA score plots at day 10 (*n* = 15 data points, 5 groups × 3 replicates) for unamplified features **(B)**, amplified features **(C)**, and Δ-features **(D)**. Replicate identifiers are labeled. Shaded ellipses represent 95% confidence regions per group. Δ-feature LDA **(D)** produced the clearest group separation at day 10, consistent with the highest LOO-CV accuracy of the three feature representations. **(E)** LOO-CV classification accuracy at the sample level (*n* = 15) for unamplified (blue), amplified (red), and Δ-feature (green) representations, shown for all timepoints pooled and for each individual timepoint. Dashed line indicates chance level (20%, five equally sized groups). LDA, linear discriminant analysis; LOO-CV, leave-one-out cross-validation; MANOVA, multivariate analysis of variance; SAA, seed amplification assay.

Wilcoxon signed-rank tests confirmed that amplification produced a significant positive delta consistently across all three features (height, width, area) in all five genotype groups (*p* = 0.004 throughout; *n* = 9 sample-timepoint pairs per group), confirming that all groups produce amplification-competent secreted material. Critically, however, the magnitude of the amplification response differed among groups in a feature- and timepoint-specific manner. Kruskal-Wallis testing of Δ Width across all timepoints pooled identified significant group differences (H = 15.28, *p* = 0.004). At Day 7, Δ Width showed a significant group effect (H = 10.43, *p* = 0.034), while at Day 10, Δ Area was significantly different among groups (H = 11.33, *p* = 0.023). Δ Width at Day 10 showed the most biologically interpretable pattern: iPD cells produced the smallest amplification-induced width increase (mean Δ Width: 0.346 log units), while *SNCA* A53T cells showed the largest (1.431), and *LRRK2* G2019S cells showed an intermediate but distinctly elevated response (1.283). Control and GBA1 N370S showed intermediate and similar values (0.798 and 0.919, respectively) (Figure 6A). When the three Δ-features were used as inputs to LDA at the sample level (*n* = 15), classification accuracy at Day 10 reached 66.7%, the highest classification performance observed across any analysis in this study, representing 3.3 times the 20% chance level, as seen on the sample-level LDA score plots at day 10 (*n* = 15 data points, 5 groups × 3 replicates) for the original unamplified features, amplified features, and Δ-features (Figures 6B–D) MANOVA on the Δ-feature vectors confirmed significant multivariate group differences at Day 10 (Pillai’s trace = 1.556, *p* = 0.014). Permutation testing confirmed this exceeded the null distribution: permutation *p*-value (10,000 permutations): *p* = 0.0002 and exact binomial test against 20% chance: *p* = 0.0001 (chance of achieving 66.67% correct classification or higher on *n* = 15 if the accuracy was due to chance only. Clopper-Pearson 95%, CI: 38.4%–88.2% (lower bound of this confidence interval still well above the 20% chance level). We note that the wide confidence interval reflects the small sample size (*n* = 15, 3 replicates per group), and the result should be interpreted as a proof-of-concept demonstration of genotype-discriminating information in the Δ-feature space rather than a validated classifier. In contrast, Δ-feature accuracy was at or below chance at Days 4 (6.7%) and 7 (6.7%), indicating that the genotype-specific signal emerges specifically during the Day 8–10 medium collection window, when each cell population has had the most time to produce mature, structurally defined extracellular aggregates. Comparison of LOO-CV accuracy at Day 10 across the three analysis conditions, unamplified/original (26.7%), amplified (46.7%), and Δ-features (66.7%), demonstrates a step-wise improvement in discriminating power, with the amplification response outperforming the amplified endpoint, which in turn outperforms the unamplified baseline (Figure 6E). This progressive improvement confirms that the structural response to amplification encodes genotype-specific biological information that is not fully captured by measuring either the pre- or post-amplification aggregate population alone.

### Staining of intracellular α-syn reveals low level of aggregation in LRRK2 G2019S neurons compared to all other groups as well as impaired clearance of α-syn in GBA1 N370S cells

**FIGURE 7:**
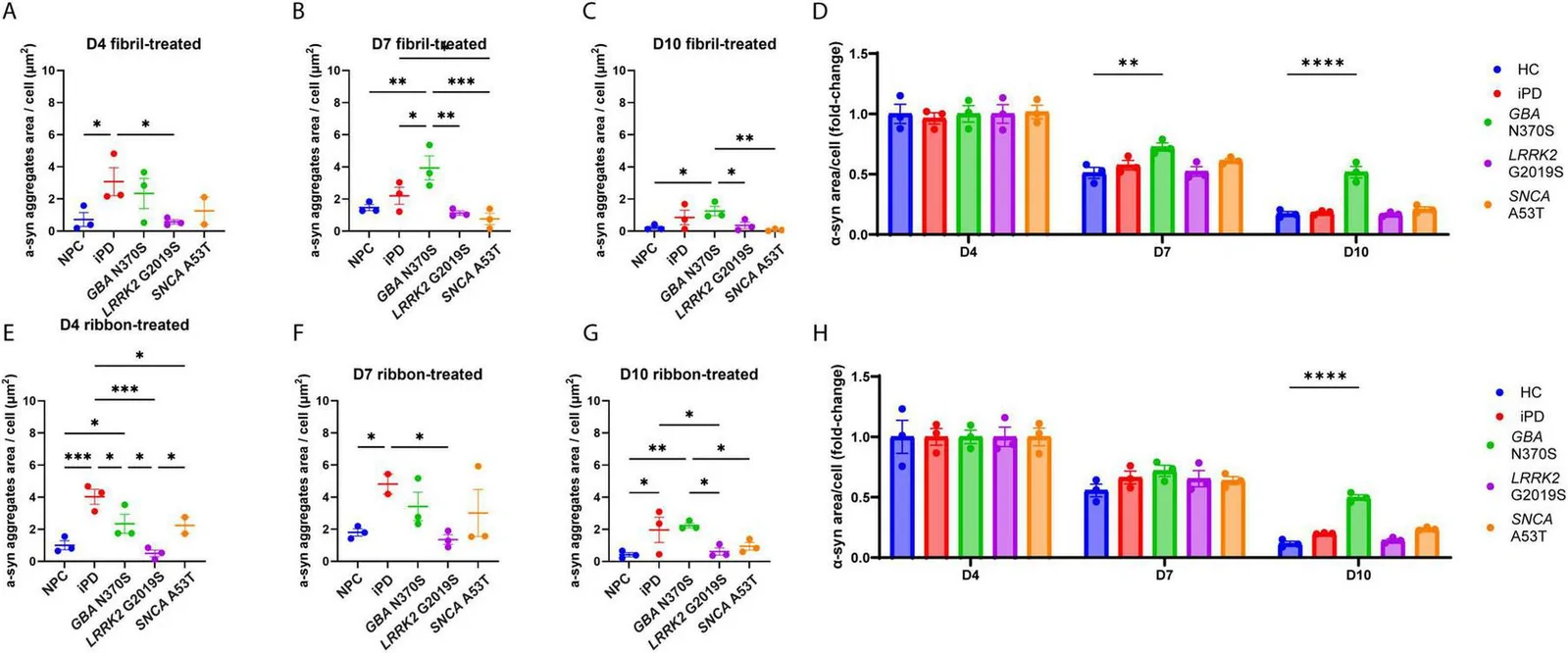
Quantification of intracellular α-syn inclusions in neurons. Differentiated iPSC-derived neurons from the five experimental groups (NPC in blue, iPD in red, *GBA1* N370S in green, *LRRK2* G2019S in purple, and *SNCA* A53T in orange) were treated with 5 μg/mL of “ribbons” or “fibrils” and subsequently fixed and stained at day 4, 7, and 10 post-treatments. Cells were immunostained for total α-syn, MAP2 and DAPI. Confocal images were taken at 40 × magnification. Three images were captured per condition and used for the analysis of α-syn inclusion area. Graphs show: α-syn area per cell (DAPI) in μm^2^ as mean ± SEM **(A–C)** and area fold-change per group/timepoint **(D)** in fibrils treated cells, and α-syn area per cell (DAPI) in μm^2^ as mean ± SEM **(E–G)** and area fold-change per group/timepoint **(H)** in ribbons-treated cells. Statistics used ordinary one-way ANOVA and two-way ANOVA. For all graphs, *P < 0.05, **P < 0.01, ***P < 0.001, ****P < 0.0001.

To compare cellular intake and α-syn inclusion formation across the different groups treated with distinct α-syn strains, “fibrils” and “ribbons” treated iPSC-derived neurons were fixed with 4% PFA and stained with total α-syn and MAP2, nuclei were counterstained with DAPI. Representative images of intracellular α-syn inclusions are included in Supplementary Figure 5. At day 4, “fibrils” treated iPD neurons showed a significant increase in α-syn inclusions area per cell compared to NPC and *LRRK2* G2019S (Figure 7A). At day 7, inclusion levels in *GBA1* N370S cells were significantly higher than the other groups (Figure 7B), and the same result was also observed at day 10 (Figure 7C), consistent with impairment in the clearance of α-syn inclusions. The iPD, *LRRK2* G2019S and *SNCA* A53T neurons did not significantly differ from NPC and all four lines had decreased α-syn inclusions over time (Figures 7A–C). Fold-change in α-syn inclusions area over the 3 timepoints was computed to better visualize α-syn clearance in each group. For both “fibrils” (Figure 7D), only the *GBA1* N370S neurons had a higher level of α-syn remaining at day 10 post-treatment. For “ribbons” treated neurons at day 4, a similar pattern emerged: iPD and *GBA1* N370S neurons had a higher area of α-syn inclusions than the NPC group, and again this was not the case for the *LRRK2* G2019S neurons (Figure 7E). However, at day 7, only iPD neurons had higher inclusions area compared to NPC, (Figure 7F). At day 10, both iPD and *GBA1* N370S neurons had higher inclusions area compared to NPC (Figure 7G). When fold-change was computed for “ribbons” (Figure 7H), we found that only the *GBA1* N370S neurons had a higher level of α-syn remaining at day 10 post-treatment, similarly to “fibrils” treated neurons. This difference in the levels of intracellular α-syn inclusions motivated a LDH cytotoxicity assay, which confirmed that there was no difference in the %cytotoxicity between the different groups at any of the experimental timepoints/treatment combinations (two-way ANOVA, Time × Group interaction: Control, *F*(5.696,14.24) = 1.63, *P* = 0.21; ribbons, *F*(7.885,19.71) = 2.00, *P* = 0.10; fibrils, *F*(7.875,19.69) = 1.45, *P* = 0.24). A modest main effect of group was detected for ribbons [*F*(4,10) = 4.03, *P* = 0.034], but Dunnett’s *post hoc* comparisons showed no individual disease group differed significantly from the NPC control at any timepoint (all adjusted *P* > 0.10) (Supplementary Figure 6).

## Discussion

In this study we investigated the role of PD-associated mutations on the cellular response to exogenous α-syn treatment with different prototypical strains. We hypothesized that in neurons treated with α-syn fibrils or ribbons, processes such as α-syn accumulation, release and strain persistence can be affected by the introduced α-syn structure and the genetic background of the neurons. Moreover, we propose that such differences may be measured by changes in α-syn species released into the CM and assessed using seed amplification and single molecule counting. We also hypothesized, based on previous published work (Bousset et al., 2013; Peelaerts et al., 2015), that strain fidelity would be preserved in the released α-syn species. iPSC-derived neurons from healthy NPC, iPD, *GBA1* N370S, *LRRK2* G2019S, and *SNCA* A53T donors were treated with α-syn variants produced under either HS buffer conditions (“fibrils”) or LS buffer conditions (“ribbons”). These two conformers have been structurally and functionally characterized, with “fibrils” exhibiting a compact, rod-like morphology with a tightly packed parallel β-sheet core and high seeding potency, while “ribbons” display a flattened, wider morphology with a distinct β-sheet arrangement and lower seeding efficiency *in vitro* (Bousset et al., 2013; Gath et al., 2014). These structural differences make them valuable tools for probing how distinct conformational templates are processed and propagated in genetically diverse neuronal backgrounds. Immunocytochemistry was performed to quantify intracellular α-syn aggregates across groups, timepoints, and treatment conditions. CM was collected to measure cytotoxicity and to detect ThT-positive secreted α-syn species, followed by SAA to investigate their amplification response and strain persistence.

A consistent feature across both treatment conditions was the strikingly low level of intracellular α-syn accumulation and ThT-positive events in CM from *LRRK2* G2019S neurons, which did not differ significantly from NPC at any timepoint. This pattern, observed for both “fibrils” and “ribbons,” suggests a fundamental impairment in the uptake or initial accumulation of exogenous α-syn in *LRRK2* G2019S neurons rather than a strain-specific effect. This is a notable finding given that *LRRK2* G2019S is associated with hyperactive kinase activity that disrupts vesicular trafficking and endolysosomal function (Cookson, 2010; West et al., 2005). While earlier *in vitro* studies reported that *LRRK2* G2019S expression can increase α-syn sequestration into inclusions over longer time courses (18 days post-exposure) (Volpicelli-Daley et al., 2016), our results within a 10-day window suggest that endocytic uptake of exogenous aggregates may itself be impaired at shorter timepoints. This interpretation is consistent with findings that *LRRK2* mutations can reduce α-syn internalization in astrocytes (Filippini et al., 2025), and with evidence that *LRRK2* G2019S impairs lysosomal proteolytic activity (Obergasteiger et al., 2020), which could alter the fate of any internalized material. CSF from *LRRK2* mutation carriers have previously shown substantially lower α-syn SAA positivity than sporadic PD patients (Siderowf et al., 2023). Moreover, recent studies have flagged α-syn oligomeric species, which are not bound to ThT, as a pathological feature of *LRRK2* PD using oligomer specific antibodies (Jensen et al., 2025; Sekiya et al., 2025). Importantly, this lack of accumulation and secretion in *LRRK2* G2019S neurons was not accompanied by increased cytotoxicity, confirming that differences in CM content are not due to cell viability effects. In contrast, iPD, *GBA1* N370S, and *SNCA* A53T neurons all showed elevated ThT-positive events in CM on days 4 and 7, followed by a gradual decline that was most pronounced in iPD and *SNCA* A53T groups. *GBA1* N370S neurons maintained persistently elevated intracellular α-syn inclusions that failed to clear over the experimental period, which may explain the corresponding elevated secreted α-syn levels in these mutation cells. This pattern is consistent with the known role of *GBA1* mutations in impairing lysosomal α-syn clearance. iPSC-derived dopaminergic neurons from *GBA1* N370S carriers exhibit autophagic perturbations and an enlarged lysosomal compartment associated with elevated extracellular α-syn (Schöndorf et al., 2014). The inverse correlation between glucocerebrosidase activity and α-syn levels measured previously in the same PPMI cell lines used in this study (Labrador-Garrido et al., 2023) provides a direct mechanistic basis for this impaired clearance phenotype. Collectively, the absence of strain-specific differences in uptake dynamics and release kinetics across all groups suggests that the gross pattern of α-syn handling (intake, accumulation, and extracellular release) is predominantly determined by the genotypic background rather than the conformational identity of the input strain.

A central finding of this study concerns the preservation of input strain characteristics in released α-syn species. Consistent with previous observations in differentiated SH-SY5Y cells (Lau et al., 2024), we found that neurons from four of the five genotype groups (NPC, iPD, *GBA1* N370S, and *LRRK2* G2019S) maintained the amplification properties of the input strain, that is ribbon-treated neurons released α-syn species with low amplification potential under our SAA conditions (55 °C for 5 h, K23Q monomer), whereas “fibril”-treated neurons released highly amplifiable species. This preservation of strain-specific amplification properties in material across distinct genetic backgrounds, including iPD neurons provides strong evidence of strain fidelity during intracellular processing and release, a process which has been previously reported (Gribaudo et al., 2019). A single exception to this pattern was observed in *SNCA* A53T neurons whereby ribbon-treated *SNCA* A53T neurons released α-syn species with amplification characteristics indistinguishable from “fibril”-treated neurons across all groups. The A53T substitution is known to alter α-syn conformation by disrupting intramolecular contacts that normally shield the NAC region from aggregation, reducing β-sheet nucleation and accelerating fibril formation kinetics *in vitro* (Conway et al., 1998; Coskuner and Wise-Scira, 2013; Stephens et al., 2020). This finding may have implications for understanding phenotypic severity in *SNCA* A53T mutation carriers. For example, if neurons constitutively amplify the seeding potency of encountered α-syn regardless of its input conformation, this could contribute to the accelerated and more severe disease course associated with *SNCA* point mutations. Further investigation is warranted to confirm strain remodeling in *SNCA* A53T neurons and to determine if other pathogenic mutations in the *SNCA* gene may have similar effects.

A key methodological contribution of this study is the demonstration that amplification-induced changes in single-particle peak features (Δ-features) provide superior genotype discrimination compared to either unamplified or amplified endpoint measurements alone. The progressive improvement in LOO-CV accuracy from 26.7% (unamplified) to 46.7% (amplified) and 66.7% (Δ-features) at Day 10, at the sample level, illustrates that the structural response to SAA, rather than the absolute aggregate characteristics before or after amplification, encodes the most biologically meaningful genotype-specific information. This finding parallels the growing use of SAA kinetic features, rather than simple endpoint measurements, to discriminate between synucleinopathies in clinical samples (Groveman et al., 2018; Rossi et al., 2025). SAA has established strong diagnostic performance for distinguishing PD from controls in CSF and other biosamples, with overall sensitivity and specificity exceeding 86% and 92%, respectively in large meta-analyses, and kinetic parameters such as time-to-threshold have been shown to carry prognostic value beyond binary positivity (Orrú et al., 2024; Wiseman et al., 2025). Our application of Δ-feature analysis to secreted aggregate species from genetically defined iPSC neurons offers an insight into how genotype-specific intracellular processing shapes the amplification competence of secreted material. The temporal evolution of discrimination, with maximal group separation emerging at Day 10 when neurons have had the longest period to impart genotype-specific modifications onto secreted aggregates, suggests that the relevant cellular processing events are slow relative to the initial period of aggregate uptake and intracellular seeding. Post-translational modifications of α-syn, including phosphorylation at Ser129, ubiquitination, and nitration, are known to alter fibril morphology and seeding behavior, and the differential activity of the autophagy-lysosome pathway and vesicular trafficking across genotype groups may progressively “stamp” secreted aggregates with distinct structural signatures over time. The finding that *SNCA* A53T and iPD neurons showed the most divergent Δ-Width responses at Day 10 is particularly intriguing, as aggregate width in our assay is proportional to hydrodynamic radius and may reflect differences in fibril length distribution and the degree of compaction of the secreted species. This may also be an indicator of secondary nucleation efficiency. The low number of ThT-positive peaks detected in unamplified conditioned medium from *LRRK2* G2019S neurons warrants consideration when interpreting Δ-features. Secreted α-syn in this genotype may exist predominantly as small oligomeric species with weak ThT binding affinity and that may not be picked up by the single-molecule detection, Furthermore, *LRRK2* G2019S-driven vesicular export may package aggregates within membrane-enclosed compartments that are inaccessible to ThT but can be disrupted during the SAA amplification protocol, releasing seed-competent material. In either case, the low unamplified peak count in *LRRK2* G2019S samples should be noted as a potential source of variance in the Δ-feature values for this genotype, as the pre-amplification baseline is computed from fewer peaks, which may contribute to the elevated Δ-feature magnitudes observed at Day 10 independently of true differences in seeding potency, and should be interpreted with caution. Future studies incorporating cryo-electron microscopy characterisation of secreted material would help resolve the structural basis for these genotype-specific Δ-feature signatures. Taken together, the results of this study demonstrate that iPSC-derived neurons from different PD genetic backgrounds respond to α-syn strain exposure in a manner that is genotype-specific at the level of aggregate uptake, intracellular accumulation, clearance kinetics, extracellular release, and critically, the structural remodeling of secreted species. The overall discrimination accuracy of 66.7% at Day 10, representing 3.3 times chance level with only 15 biological replicates, is encouraging given the relatively small sample size and the complexity of the experimental design. Larger studies incorporating additional cell lines per genotype, longer observation windows, and complementary structural characterisation of secreted aggregates would be expected to substantially improve discrimination. The Δ-feature analysis framework introduced here, using the amplification response as a probe of genotype-specific seeding potency, may be applicable to patient-derived biofluids in future clinical studies, particularly in the context of genotype-stratified SAA analyses for early diagnosis or patient stratification in clinical trials.

Several limitations of this study warrant consideration. The relatively short 10-day observation window following α-syn treatment captures early events in aggregate processing but may not fully reflect the longer-term dynamics of strain remodeling and secretion relevant to disease progression over years. The analysis was restricted to “fibrils”-treated neurons for the LDA discrimination analysis due to the low amplification signal from “ribbons”-treated groups, which precluded direct comparison of strain-specific effects on Δ-feature discrimination. Buffer conditions such as pH and salt concentrations are known to affect the amplification of different α-syn species. It has previously been shown that “ribbons” can amplify more readily in acidic buffer (Lau et al., 2024) offering a way to investigate amplification response differences in our different groups for “ribbons” treated cells. Moreover, the use of *n* = 3 biological replicates per group, while consistent with constraints of the iPSC field and adequate for preliminary discrimination analysis, limits the statistical power of sample-level analyses.

A theoretical limitation of the K23Q amplification approach is that the substituted monomer substrate may not template equally with all input seed conformations. Should K23Q exhibit an intrinsic preference for propagating fibril-like rather than ribbon-like structural properties, the observed asymmetry in amplification potency between HS fibril- and LS ribbon-treated conditions could in part reflect substrate bias rather than exclusively reporting on the seeding competence of secreted aggregates. This possibility would be best addressed in future work by comparing K23Q amplification products seeded with structurally characterized reference fibrils and ribbons using cryo-electron microscopy or solid-state NMR, to establish whether single-molecule peak morphology features are faithfully propagated from input seed to amplification product for both polymorphs. Importantly, however, two observations in the present dataset are consistent with genuine cellular milieu and/or seed-dependent rather than purely substrate-driven amplification. First, the genotype differences observed between the genotype groups before amplification which are consistent with known biology of each mutation group as discussed above. Second, the divergence of the LS-ribbons amplification response in *SNCA* A53T from the other genotype groups which is hard to explain by fixed substrate bias but is instead consistent with active cellular remodeling of the input strain conformation prior to secretion.

Another limitation of this study is that ThT fluorescence reports generic amyloid cross-β structure rather than α-synuclein specifically. ThT and related compounds are known to bind other amyloidogenic species, including tau neurofibrillary tangles and amyloid-β plaques. Moreover, heterologous cross-seeding between amyloidogenic proteins including between α-synuclein and tau, and between amyloid-β and α-synuclein has been reported (Bassil et al., 2020; Williams et al., 2020; Zhan et al., 2025). It therefore remains possible that non-α-synuclein amyloidogenic material contributed to, or cross-seeded, the ThT signal measured in our assay. In this study we did not perform direct biochemical measurement of tau or amyloid-β (e.g., western blot, ELISA) in conditioned media since simply detecting these proteins would not establish their aggregation state or cross-seeding potential. Thus, the extent to which ThT fluorescence associates only with alpha-synuclein under the conditions employed remains to be determined. However, several features of our data support that the alpha-synuclein is likely the predominate source of ThT fluorescence. First, our assay reports amplification of an exogenously supplied recombinant α-synuclein monomer substrate rather than direct ThT staining of the media itself, so any contribution from tau or amyloid-β would require these species to effectively cross-seed this substrate under reaction conditions optimized for homologous α-synuclein template propagation. Heterologous amplification is a comparatively inefficient process relative to seeding by the native protein. For example, amyloid-β and other fibril preparations failed to trigger amplification of recombinant α-synuclein monomer under alpha-synuclein seed amplification conditions (Mao et al., 2024). It was also observed that ribbon-conditioned media generated substantially weaker ThT signal than fibril-conditioned media despite both being derived from the same neuronal system and processed identically. This polymorph-dependent difference may not be observed if signal were driven by generic, seed-independent amyloid contamination in the media. Finally, ThT fluorescence rose substantially above assay baseline in fibril-seeded reactions, which is not readily explained by low-level non-specific aggregated protein carried over in the conditioned media. Nonetheless, future work using mass spectrometry-based characterisation of the amplified aggregate, or parallel non-α-synuclein SAAs run on the same media, would further exclude a contribution from non-native amyloid species.

Additionally, an important limitation of the current study is that the differences observed can only be used to describe the specific mutations occurring in our cell lines, and may not extend to other mutations in these genes. Similarly, caution should be used in extrapolating results from the specific α-syn polymorphs used in this study to other potential occurring strains and mixes of strains. Likewise, it is unknown whether the results obtained in this study are cell type specific and other outcomes may occur for example with dopamine or glia. Finally, while LDH cytotoxicity measurements confirmed no group differences in cell viability, more sensitive assays of neuronal health and synaptic integrity would strengthen the conclusion that differences in secreted α-syn properties reflect active cellular processing rather than passive leakage. Different outcomes may also occur if more mature neurons are employed. Despite these limitations, this study provides the first systematic evidence that PD-associated genetic mutations differentially affect the amplification-response of secreted α-syn in a manner detectable at the single-particle level and establishes the Δ-feature analysis of SAA-amplified conditioned medium as a promising tool for probing genotype-specific α-syn biology.

## Data Availability

The raw data supporting the conclusions of this article will be made available by the authors, without undue reservation.

## Footnotes

1
https://www.ppmi-info.org/access-data-specimens/request-cell-lines
