# Supplementary material for: Seed amplification of released alpha-synuclein strains reveals genotype-specific differences in Parkinson’s disease neurons
Source: Front Aging Neurosci. 2026 Sep 8;18:1917981. doi: 10.3389/fnagi.2026.1917981 (PMC13593840; doi:10.3389/fnagi.2026.1917981)
Supplement: Supplementary file 1 [file Data_Sheet_1.pdf]

S1

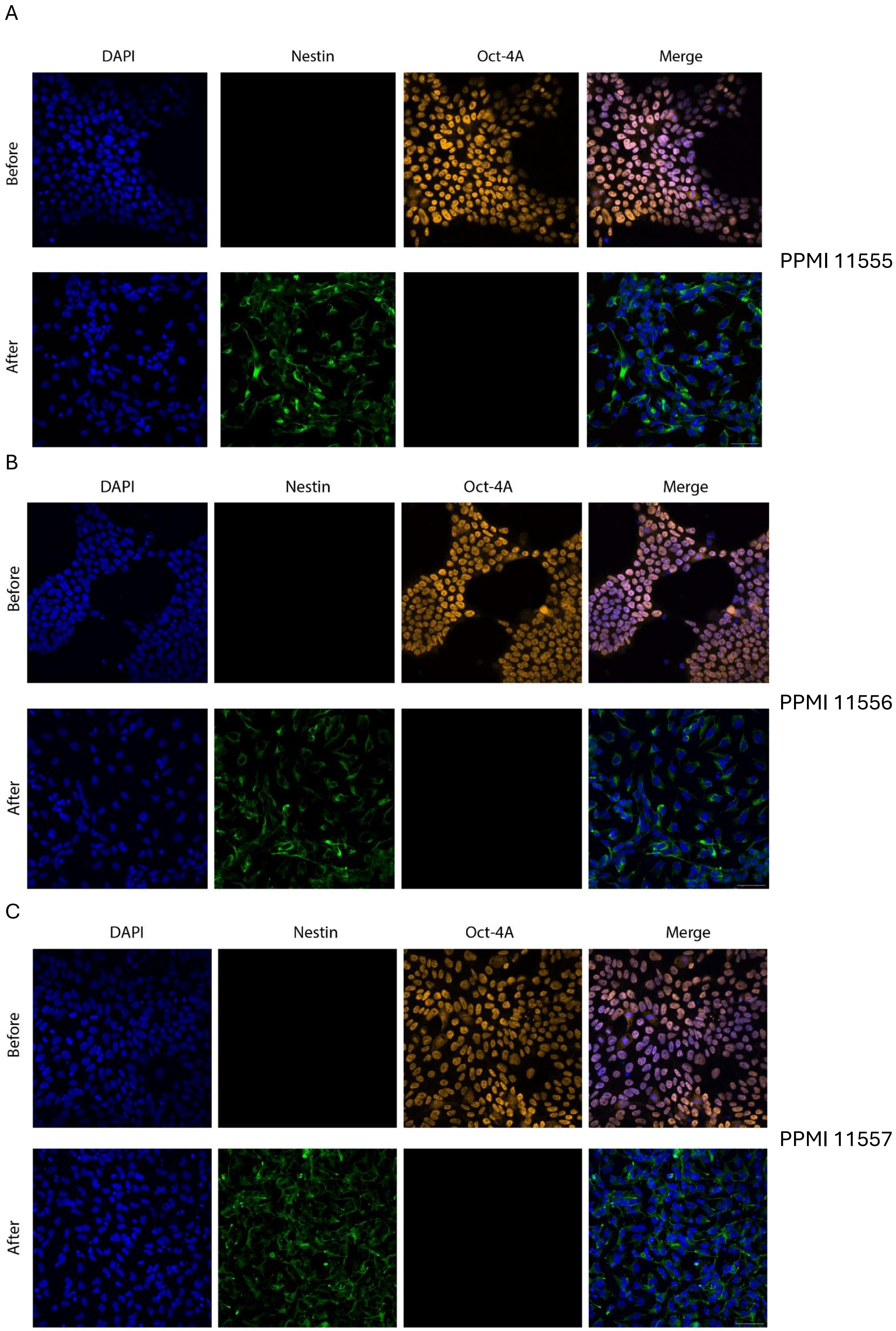
A
DAPI
Nestin
Oct-4A
Merge
Before
After
PPMI 11555
B
DAPI
Nestin
Oct-4A
Merge
Before
After
PPMI 11556
C
DAPI
Nestin
Oct-4A
Merge
Before
After
PPMI 11557

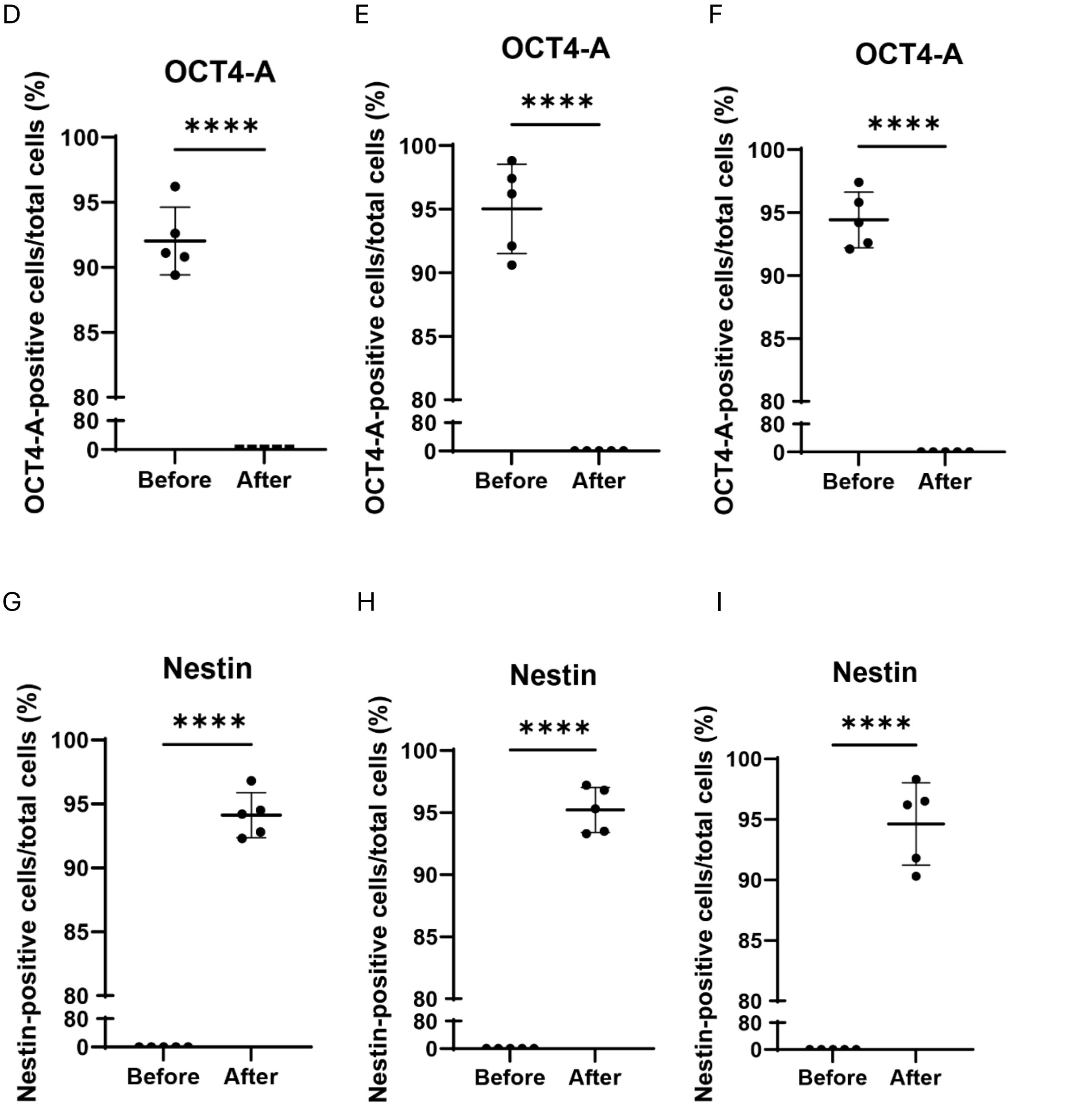

Supplementary figure 1- Characterization of SNCA A53T cells with IPSCs and NSCs markers. IPSCs from 3 SNCA A53T cell lines (PPMI 11555, 11556 and 11557) were stained with ISPC marker OCT-4 and NSC marker Nestin before and after 7-day neural induction. Cells were fixed, stained, and imaged using a Nikon C2 confocal microscope at 40x magnification. Images were analysed using ImageJ, and the percentage of OCT-4 and Nestin expressing cells was calculated. Representative images from cell line PPMI 11555 (A), 11556 (B) and 11557 (C), before and after neural induction. Channels shown are DAPI (blue), Nestin (green) and OCT-4 (orange). Percentage of OCT-4 expressing cells (D-F) and Nestin expressing cells (G-I) for each of the 3 lines, respectively. Graph shows OCT4- or Nestin-positive cells/total cells as mean ± SD (n=5). Statistics used unpaired t-test. For all graphs, ****P < 0.0001.

S2

A

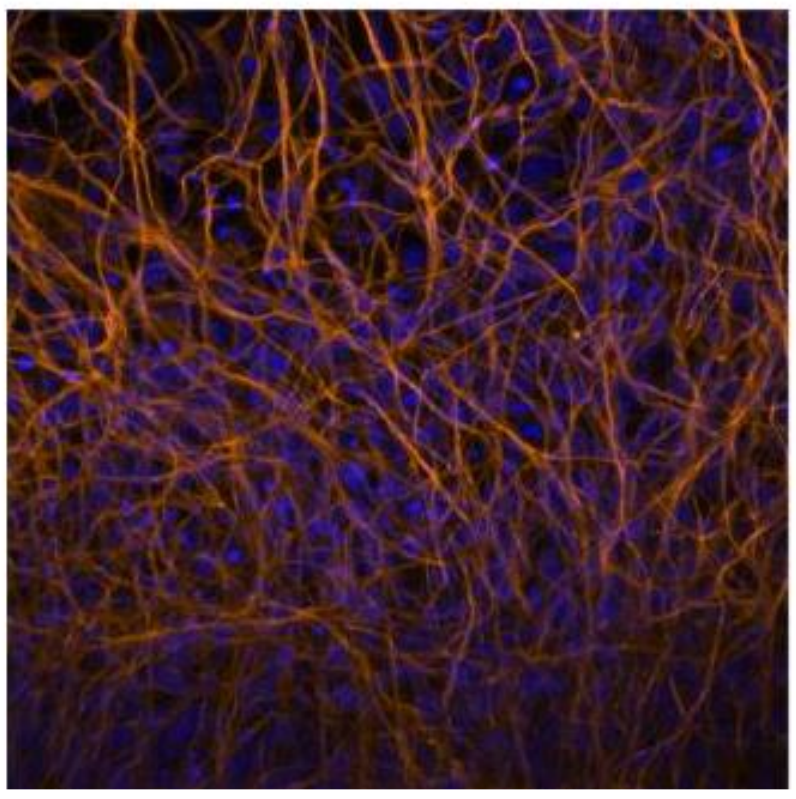

B

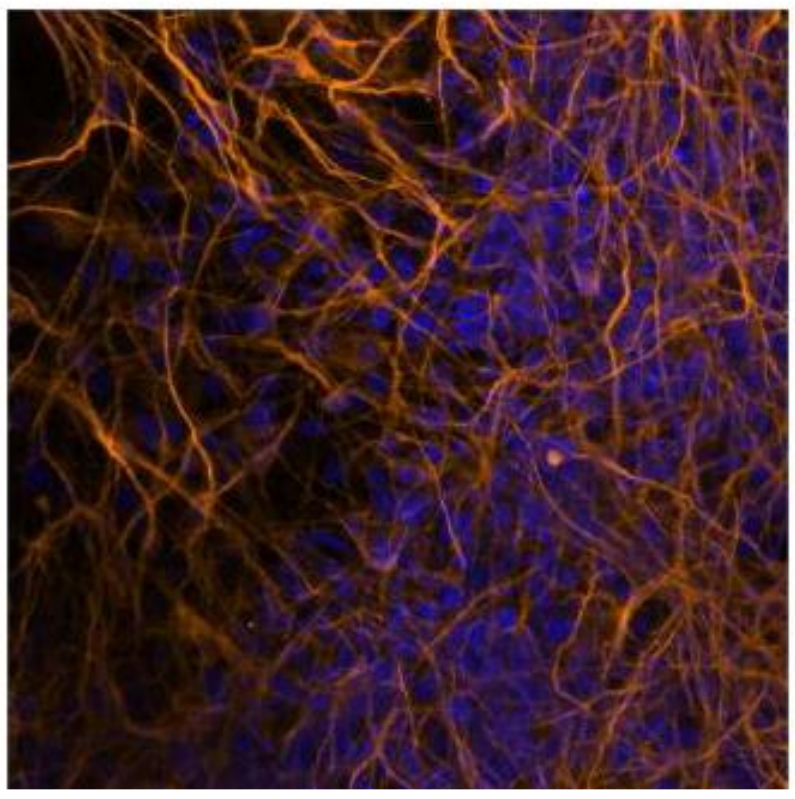

C

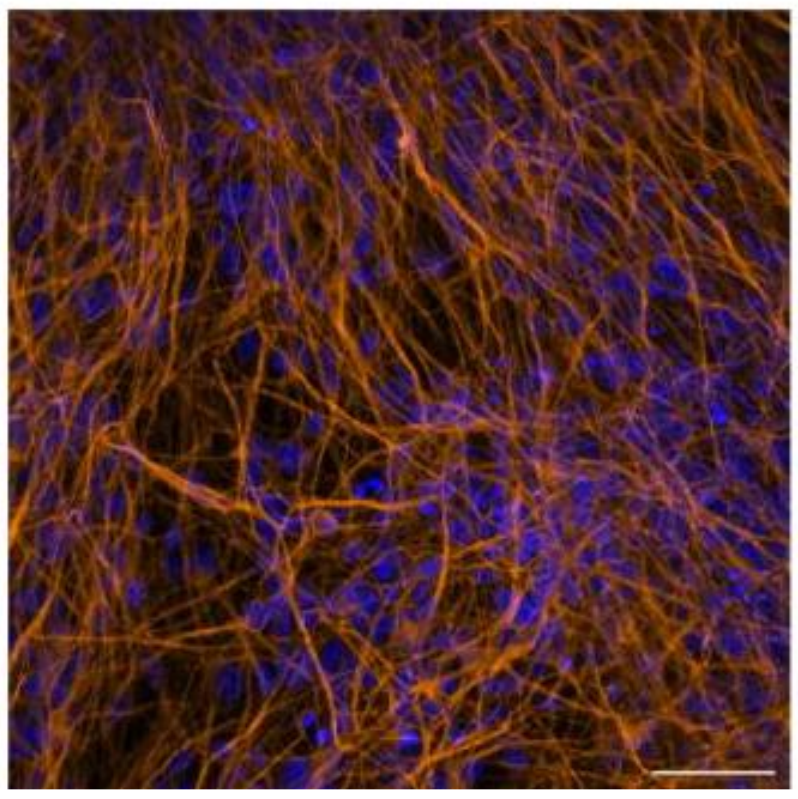

PPMI 11555 PPMI 11556 PPMI 11557

D

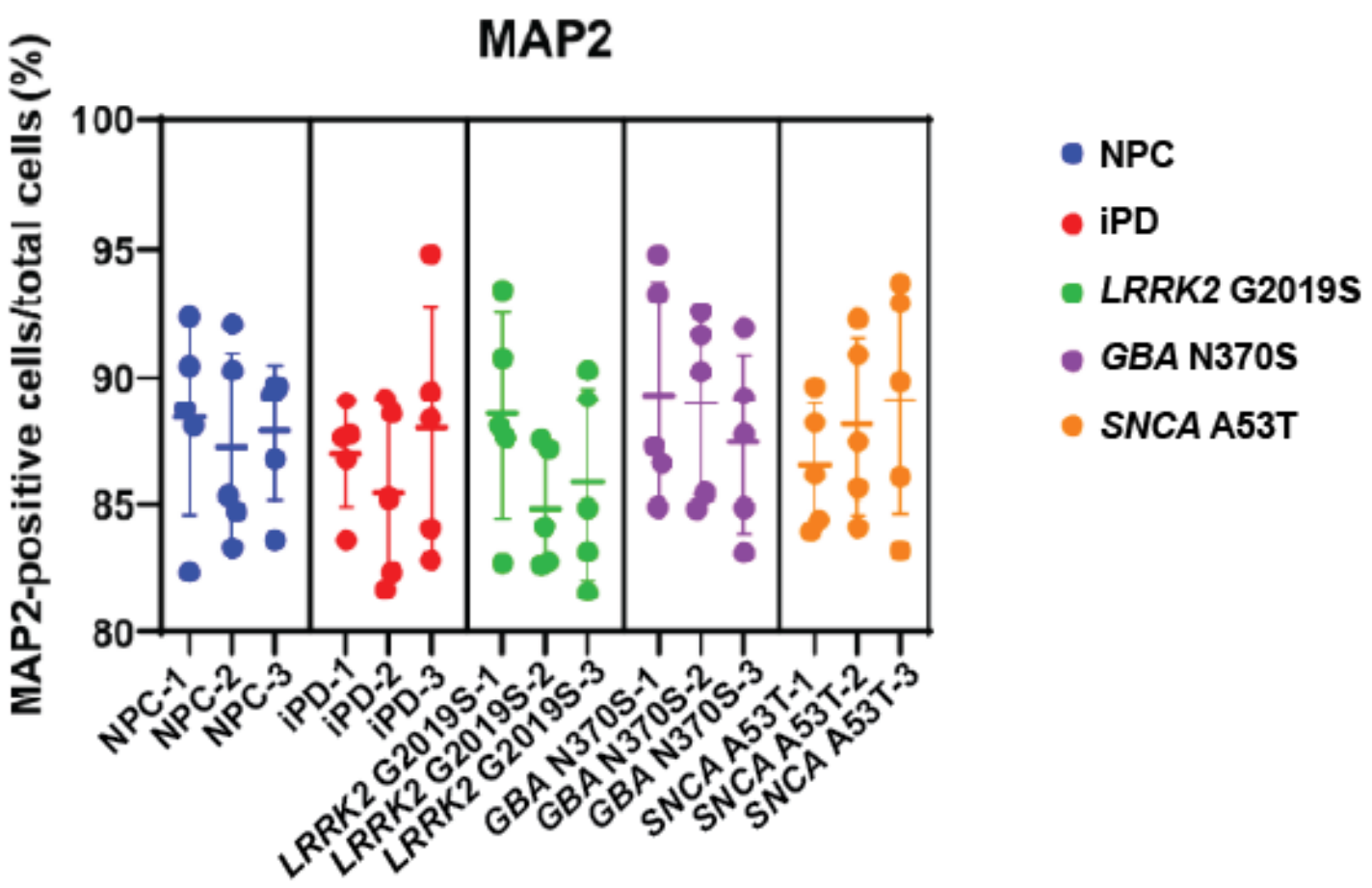

E

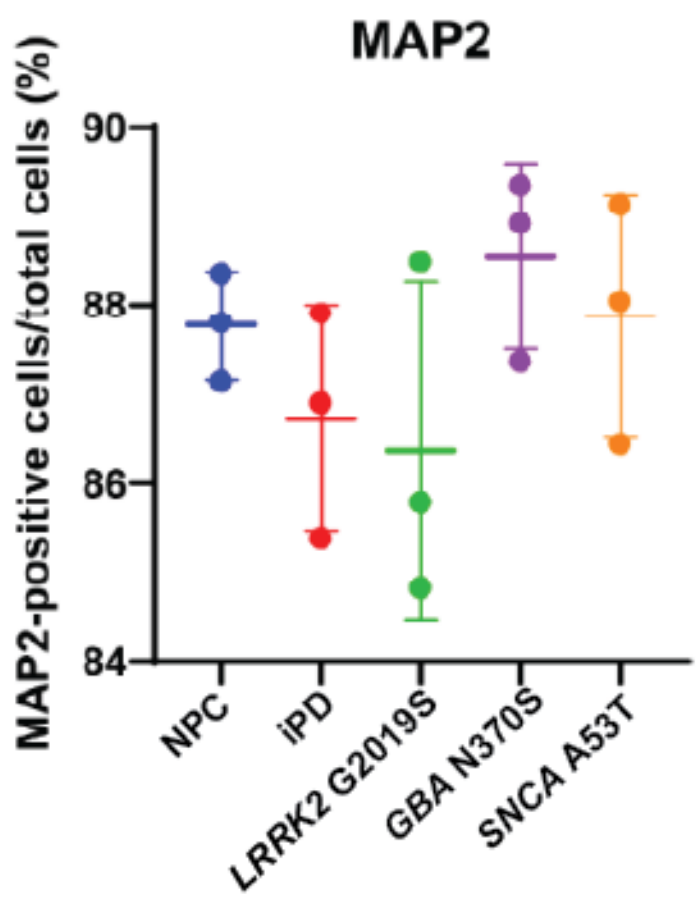

Supplementary figure 2- Staining of SNCA A53T cells with neuronal marker MAP2 and comparison with other genotype groups. NSCs from 3 SNCA A53T cell lines (PPMI 11555, 11556 and 11557) were differentiated into mature neurons and stained with neuronal marker MAP2. Cells were fixed, stained, and imaged using a Nikon C2 confocal microscope at 40x magnification. Images were analysed using ImageJ, and the percentage of MAP2 expressing cells was calculated. Representative images from cell line PPMI 11555 (A), 11556 (B) and 11557 (C) after neural differentiation. Channels shown are DAPI (blue) and MAP2 (orange). Graphs show the percentage of MAP2 expressing cells in each of the 15 cell lines (3 lines per genotype group) (D) and in each genotype group (E). Data is shown as mean ± SD.

S3 A

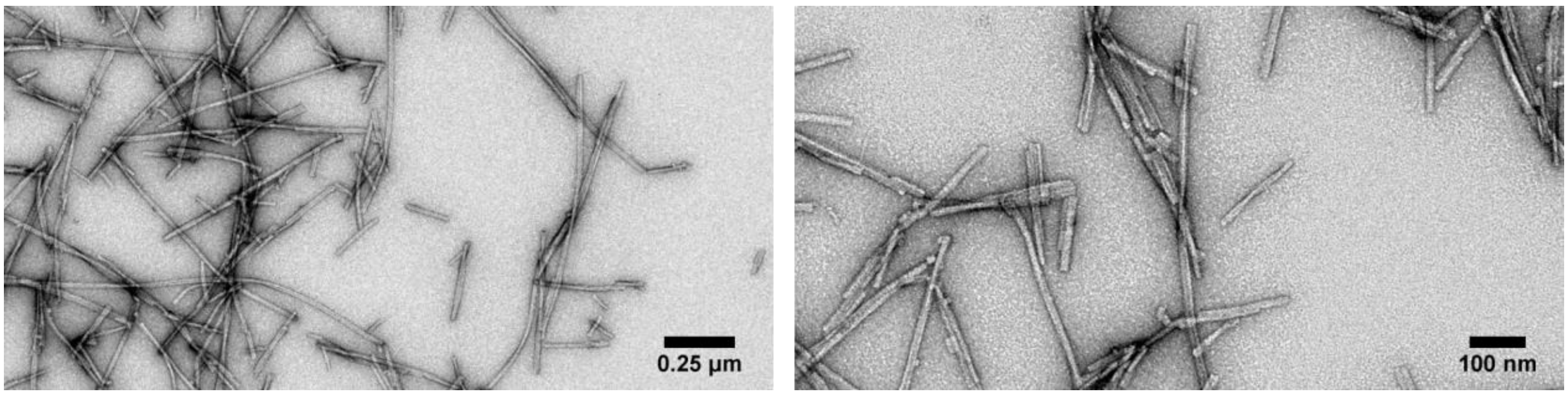

B

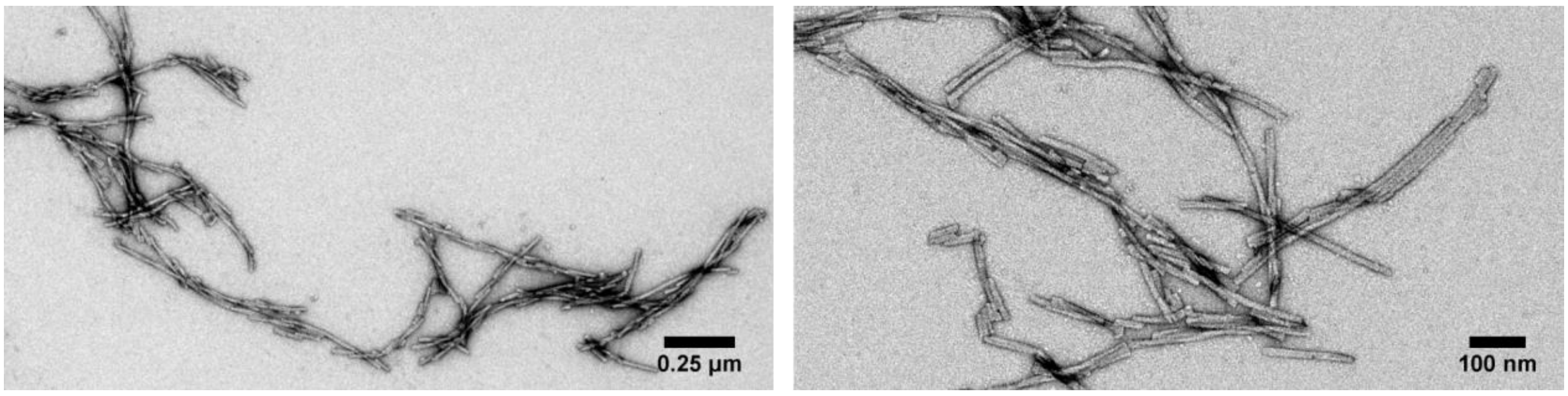

Supplementary figure 3- Transmission electron microscopy of α-Synuclein ribbons and fibrils. Representative negative-stain transmission electron microscopy image obtained for Ribbons (A) and fibrils (B). Ribbons display a characteristic flattened morphology with occasional twists; scale bar as indicated. Fibrils display long, comparatively thin cylindrical filaments, with twisting visible along some structures; scale bar as indicated. Samples were adsorbed for 1 min onto glow-discharged, carbon/Formvar-coated 200-mesh copper grids. Excess sample was wicked away, and the grids were stained with 2% (w/v) uranyl acetate for 30 s. Staining was repeated twice before the grids were air-dried and imaged using an FEI Tecnai G2 20 TEM at 38,000× magnification.

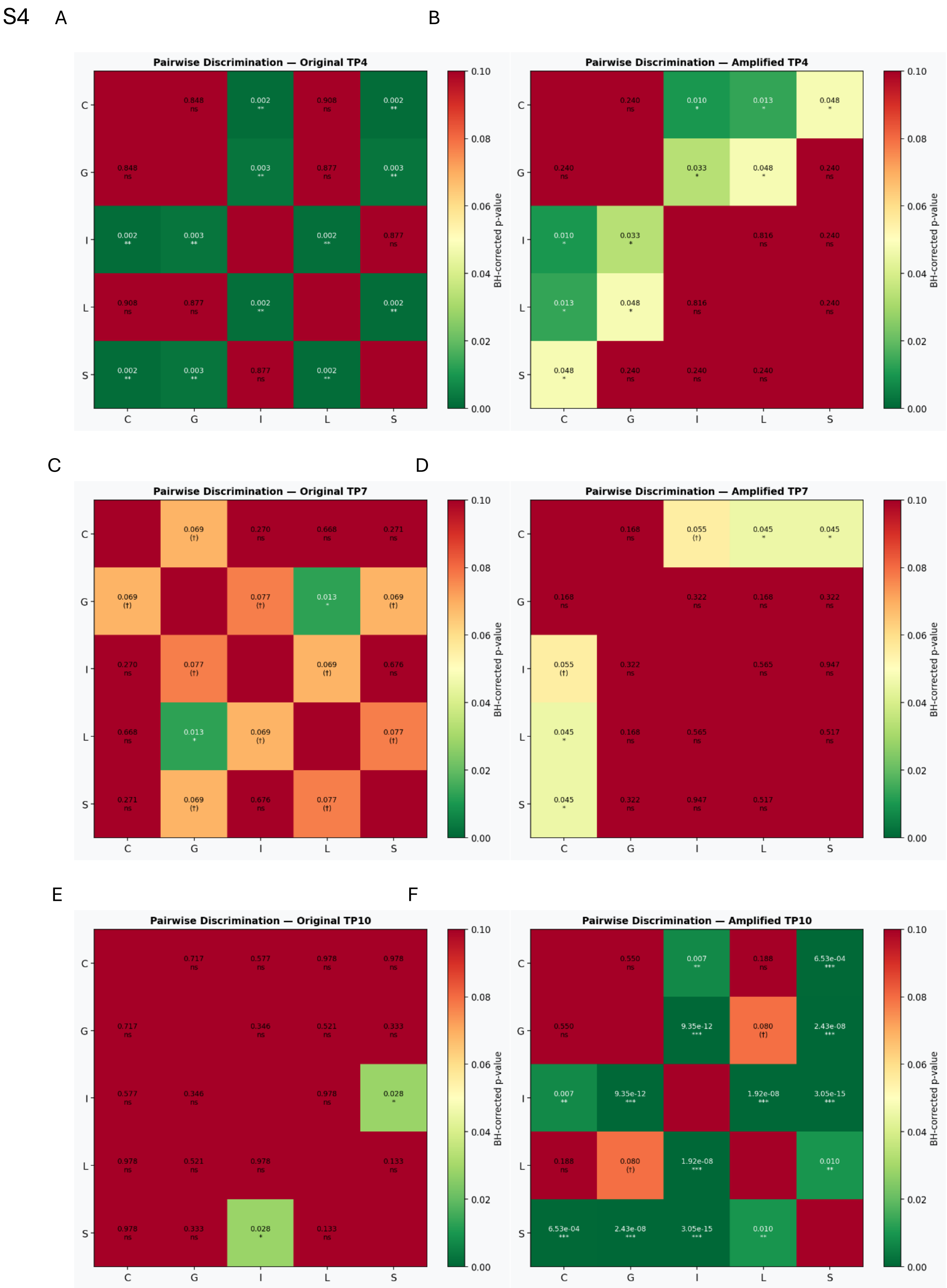

Supplementary figure 4- Pairwise genotype group discrimination heatmaps based on LDA of single molecule morphology features in original and amplified CM collected at different timepoints. Group-dsicrimination was performed for each timepoint seperately in original CM collected at day 4 (A), day 7 (C) and day 10 (E), and in the amplified CM collected at day 4 (B), day 7 (D) and day 10 (F). Pairwise discrimination was assessed by Mann-Whitney U tests applid to scores along LD1. All p-values were adjusted for multiple comparisons across 10 pairwise te sts using the Benjamini-Hochberg (BH) false discovery rate procedure. Colour-scale represents the BH-corrected P-value and is annotated within each heatmap. Ns, not significant, *P<0.05, **P<0.01, ***P<0.001.

S5

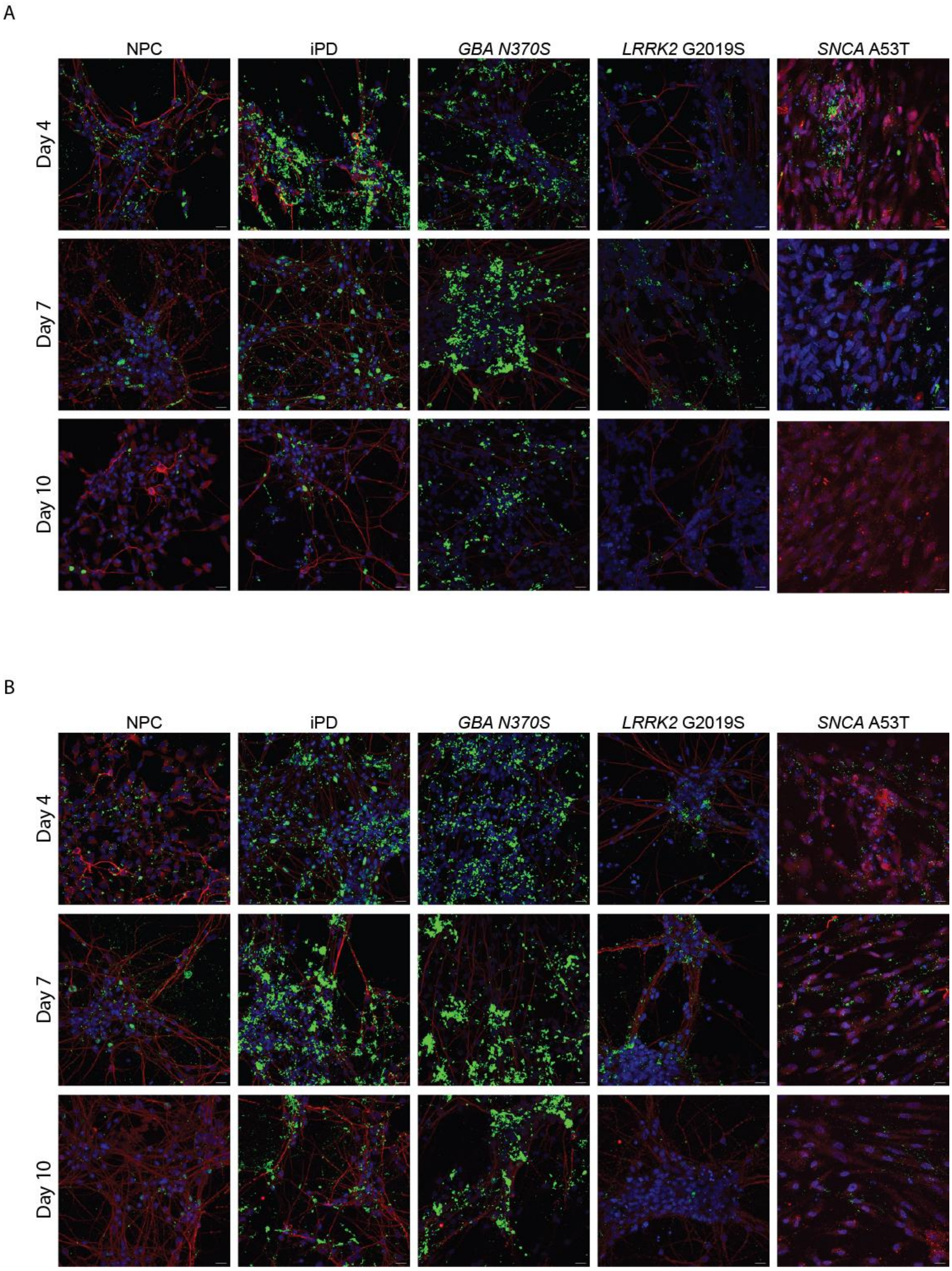

Supplementary figure 5- Intracellular α-syn inclusions in "fibrils" (A) and "ribbons" (B) treated neurons from the five experimental groups. Differentiated iPSC-derived neurons were treated with 5 μg/mL of "ribbons" and "fibrils". Neurons were subsequently fixed for immunofluorescence staining on Day 4, 7, and 10 post-treatment. (A) Cells were fixed and stained for total α-syn (green), MAP2 (red) and DAPI (blue). Confocal images were taken at 40x magnification. Scale bar is 20 μm. Three images were captured per condition and used for the analysis of α-syn inclusions area. One representative image per group / timepoint / treatment is shown above.

S6

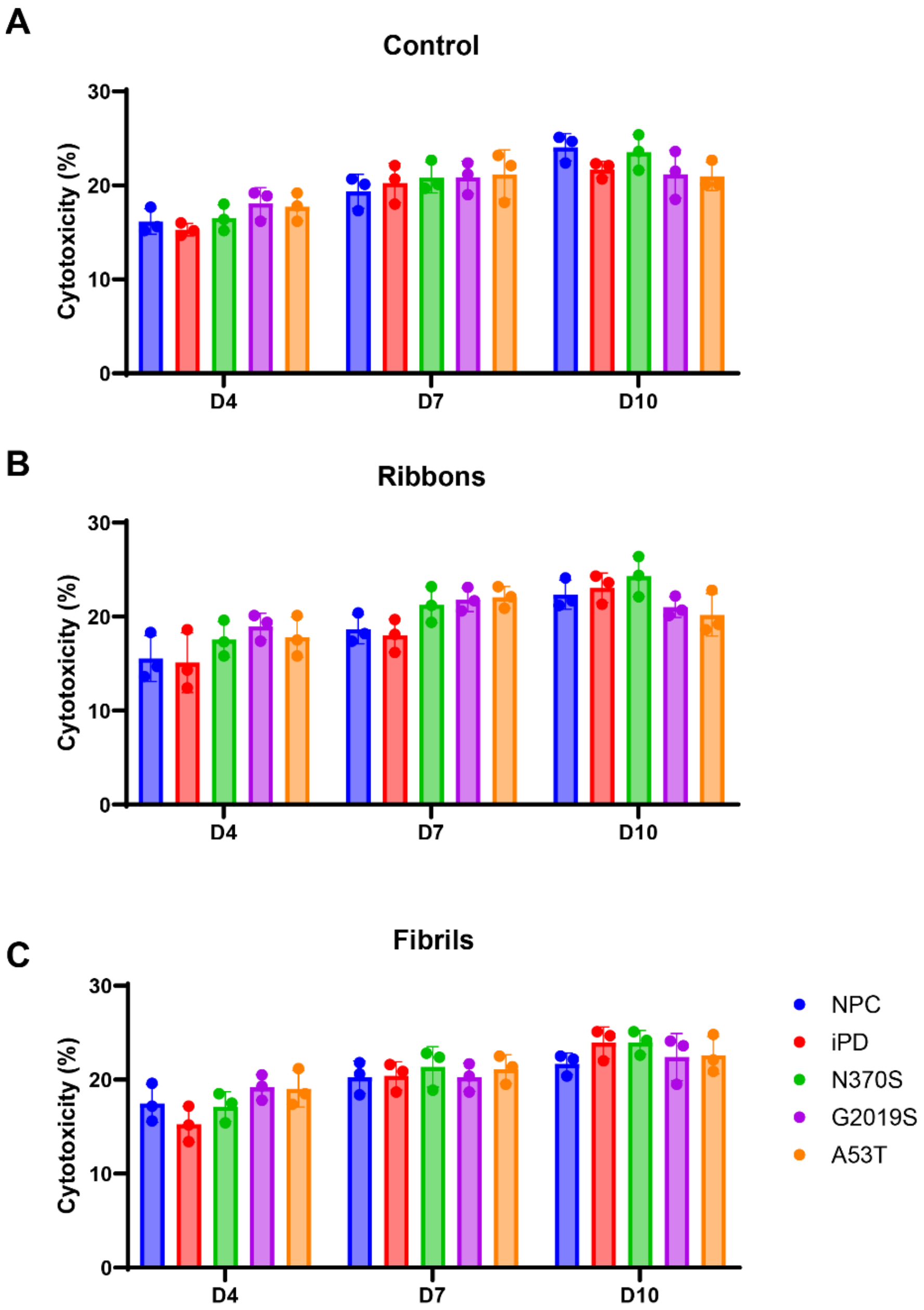

Supplementary figure 6- Comparison of cytotoxicity between study groups. Cytotoxicity of treatment with exogenous α-syn assemblies in iPSC-derived neurons. Graphs show cytotoxicity in neurons after incubation with "ribbons" (B), "fibrils" (C) at concentrations of 5 µg/mL. Cells treated with PBS serve as the control (A). LDH released in cells was determined using the colorimetric cytotoxicity assay after treatment. Cytotoxicity was assessed at 4, 7 and 10 days after treatment. Graphs were plotted by different study groups: NPC (blue), iPD (red), *GBA* N370S (green), *LRRK2* G2019S (purple), *SNCA* A53T (orange). Bar graph of average percentage of cytotoxicity to maximum lactate dehydrogenase release. Error bars represent mean ± SD (n =3 biological replicates, each representing the average of 3 technical replicates). Statistics used ordinary two-way ANOVA.
