# Supplementary material for: Seed amplification of released alpha-synuclein strains reveals genotype-specific differences in Parkinson’s disease neurons
Source: Front Aging Neurosci. 2026 Sep 8;18:1917981. doi: 10.3389/fnagi.2026.1917981 (PMC13593840; doi:10.3389/fnagi.2026.1917981)
Supplement: Supplementary file 2 [file Data_Sheet_2.pdf]

Appendix: Python script of Single Molecule analysis

```
# -*- coding: utf-8 -*-
"""
Created on Fri May 22 10:22:58 2020
@author: ygamb
"""
####################################################################
#######
## Librairy
####################################################################
#######
import matplotlib.pyplot as plt
import numpy as np
import matplotlib.image as mpimg
import os
from skimage import exposure
from scipy.ndimage import gaussian_filter
from skimage.morphology import reconstruction
import pandas as pd
# import cv2
from skimage import color
from os import chdir
import scipy.signal as signal
from scipy.signal import butter,filtfilt
from matplotlib.colors import LogNorm
import re
####################################################################
#######
## Recuperation des fichiers
####################################################################
#######
c = os.getcwd()
repertoire = os.walk(c)
liste_elem = []
for root, dirs, files in repertoire :
liste_elem.append([root, files])
####################################################################
#######
## Traitement des donnees
####################################################################
#######
def butter_lowpass_filter(data, cutoff, fs, order):
normal_cutoff = cutoff / nyq
# Get the filter coefficients
b, a = butter(order, normal_cutoff, btype='low', analog=False)
y = filtfilt(b, a, data)
return y
```



```
####################################################################
#######
## reccuperer les pics pics
####################################################################
#######
##Step 1 : Define the filter requirements
```

```
# Filter requirements.
T = 3000 # Sample Period
fs = 1000.0 # sample rate, Hz
cutoff = 70 # desired cutoff frequency of the filter, Hz , slightly
higher than actual 1.2 Hz, lower means more stringent (remove more
peaks) with higher number indicating less stringent (will include a
lot of peaks)
nyq = 0.5 * fs # Nyquist Frequency
order = 2 # sin wave can be approx represented as quadratic
n = int(T * fs) # total number of samples
rapport = 5
ref1 = 0
taille = 120 # Calcul ecart type
mean_intensity = []
mean_width = []
mean_intengral = []
liste_nbr_peak = []
liste_name = []
liste_rapp = []
liste_mean_fin = []
#Step 2 : Filter implementation using scipy
for elem in liste_elem :
chdir(elem[0])
liste_fichier = elem[1]
for fichier in liste_fichier :
if fichier[len(fichier)-5:] != ".xlsx" and
fichier[len(fichier)-4:] != ".png" and fichier[len(fichier)-4:] !=
".csv" and fichier[len(fichier)-3:] != ".py" and
fichier[len(fichier)-4:] != ".txt":
liste = pd.read_table(fichier)
label = liste.columns[0]
liste_y = liste[label].tolist()
y = butter_lowpass_filter(liste_y, cutoff, fs, order)
liste_x = np.linspace(0,len(liste_y)-1,len(liste_y))
liste_prom = []
liste_mean = []
for i in range (1,len(y)-taille,taille):
liste_prom.append(np.std(liste_y[i:i + taille])*rapport)
liste_mean.append(np.mean(liste_y[i:i + taille]))
prominence_min = min(liste_prom)
back = liste_mean[liste_prom.index(prominence_min)]
liste_mean_fin.append(back)
bruit = back/(prominence_min/rapport)**2
peaks, properties = signal.find_peaks(y, prominence
=(prominence_min,None))
168
widths = signal.peak_widths(y, peaks, rel_height=0.5) # rel_height
define evaluation height of a peak to determine the width of peak.
At a 0.5, it is 50% of prominence so you are obtaining width at half
height of peak (above background). 0.5 gives the FWHM
liste_p = []
for p in peaks :
liste_p.append(y[p])
liste_left = widths[2]
```

```
liste_right = widths[3]
liste_intensite = []
liste_integrale = []
for i in range(len(peaks)) :
left = int(liste_left[i])
right = int(liste_right[i])
liste_integrale.append(sum(liste_y[left:right])-
len(liste_y[left:right])*back)
liste_intensite.append(max(liste_y[left:right])-back)
fig = plt.figure(figsize=(50,30))
plt.plot(liste_x,liste_y, label = "prominence : " +
str(prominence_min))
plt.plot(liste_x,y, label = "background : " + str(back))
plt.legend(fontsize = 60)
plt.ylabel("Intensity", fontsize = 60)
plt.xlabel("Time (ms)", fontsize = 60)
plt.title(fichier, fontsize = 80)
plt.tick_params(labelsize = 40)
if bruit >= ref1 :
plt.plot(peaks, liste_p, 'o', markeredgecolor = "r", markersize =
20, label = "rapport : " + str(bruit))
plt.legend(fontsize = 60)
data = [[len(peaks)],
peaks,
liste_intensite,
widths[0],
liste_integrale,
[back],
[bruit]]
df = pd.DataFrame(data, index = ['Peak number',
'Peak position',
'Peak intensity',
'Peak width',
'Peak intengrale',
'Background',
'Rapport'])
if len(peaks)>0 :
mean_intensity.append(sum(liste_intensite)/len(peaks))
mean_width.append(sum(widths[0])/len(peaks))
mean_intengral.append(sum(liste_integrale)/len(peaks))
liste_nbr_peak.append(len(peaks))
else :
mean_intensity.append(None)
mean_width.append(None)
mean_intengral.append(None)
liste_nbr_peak.append(len(peaks))
else :
169
plt.axhline(np.mean(liste_y), linestyle= '--', color = 'r', label =
'Mean ='+ str(np.mean(liste_y)))
plt.legend(fontsize = 60)
data = [[np.mean(liste_y)], [prominence_min/rapport],
[sum(liste_y)], [bruit]]
df = pd.DataFrame(data, index = ['Mean',
```

```
'Std',
'Integral',
'Rapport'])
mean_intensity.append(np.mean(liste_y))
mean_width.append(None)
mean_intengral.append(sum(liste_y))
liste_nbr_peak.append("High concentration")
liste_rapp.append(bruit)
liste_name.append(fichier)
df.to_csv(fichier+".csv")
plt.savefig(fichier+".png")
plt.show()
# plt.hist(liste_integrale, 20)
# plt.title("Integrale : " + fichier)
# plt.savefig(fichier+"_hist integrale.png")
# plt.show()
# plt.hist(liste_intensite, 20)
# plt.title("Intensitee : "+ fichier)
# plt.savefig(fichier+"_hist intensite.png")
# plt.show()
chdir(liste_elem[0][0])
data = {'Name' : liste_name,
'Peak number' : liste_nbr_peak,
'Mean peak intensity' : mean_intensity,
'Mean peak width' : mean_width,
'Mean peak intengrale' : mean_intengral,
'background' : liste_mean_fin,
'Rapport' : liste_rapp}
df = pd.DataFrame(data)
df.to_csv("final_analyse.csv")
liste_fich = np.linspace(0,len(liste_rapp)-1,len(liste_rapp))
fig = plt.figure(figsize=(50,30))
plt.plot(liste_fich,liste_rapp, 'o', markeredgecolor = "r",
markersize = 30)
plt.axhline(ref1, linestyle= '--', color = 'b', label = 'rapport ='+
str(ref1))
plt.legend(fontsize = 60)
plt.ylabel("Rapport", fontsize = 60)
plt.xlabel("Files", fontsize = 60)
plt.title("Rapport", fontsize = 80)
plt.tick_params(labelsize = 40)
plt.savefig("Rapport.png")
plt.show()
170
fig = plt.figure(figsize=(50,30))
plt.plot(liste_fich,liste_mean_fin, 'o', markeredgecolor = "r",
markersize = 30)
plt.legend(fontsize = 60)
plt.ylabel("Mean", fontsize = 60)
plt.xlabel("Files", fontsize = 60)
plt.title("Mean", fontsize = 80)
plt.yscale("log")
plt.tick_params(labelsize = 40)
plt.savefig("Mean.png")
```

```
plt.show()
#
##############################################################################
# ## Sauvegarder la taille des images dans un fichier excel
#
##############################################################################
# L = blobs_dog
# dfObj = pd.DataFrame(L)
# dfObj.to_csv("fichier_ref.csv")
```
